# Loss of polycomb repressive complex 1 activity and chromosomal instability drive uveal melanoma progression

**DOI:** 10.1038/s41467-021-25529-z

**Published:** 2021-09-13

**Authors:** Mathieu F. Bakhoum, Jasmine H. Francis, Albert Agustinus, Ethan M. Earlie, Melody Di Bona, David H. Abramson, Mercedes Duran, Ignas Masilionis, Elsa Molina, Alexander N. Shoushtari, Michael H. Goldbaum, Paul S. Mischel, Samuel F. Bakhoum, Ashley M. Laughney

**Affiliations:** 1 Department of Ophthalmology and Visual Science, Yale University School of Medicine, New Haven, CT USA; 2 Department of Ophthalmology, University of California San Diego, La Jolla, CA USA; 3 Department of Surgery, Memorial Sloan Kettering Cancer Center, New York, NY USA; 4 Department of Ophthalmology, Weill Cornell Medical School, New York, NY USA; 5 Human Oncology and Pathogenesis Program, Memorial Sloan Kettering Cancer Center, New York, NY USA; 6 Department of Pharmacology, Weill Cornell Graduate School, New York, NY USA; 7 Institute for Computational Biomedicine, Weill Cornell Medicine, New York, NY USA; 8 Department of Physiology and Biophysics, Weill Cornell Medicine, New York, NY USA; 9 Sandra and Edward Meyer Cancer Center, Weill Cornell Medicine, New York, NY USA; 10 The Alan and Sandra Gerry Metastasis and Tumor Ecosystems Center, Memorial Sloan Kettering Cancer Center, New York, NY USA; 11 Sanford Consortium for Regenerative Medicine, University of California San Diego, La Jolla, CA USA; 12 Department of Medicine, Memorial Sloan Kettering Cancer Center, New York, NY USA; 13 Department of Medicine, Weill Cornell Medical School, New York, NY USA; 14 Department of Pathology, Stanford University School of Medicine, Stanford, CA USA; 15 ChEM-H, Stanford University, Stanford, CA USA; 16 Department of Radiation Oncology, Memorial Sloan Kettering Cancer Center, New York, NY USA

**Keywords:** Eye cancer, Metastasis, Tumour heterogeneity

## Abstract

Chromosomal instability (CIN) and epigenetic alterations have been implicated in tumor progression and metastasis; yet how these two hallmarks of cancer are related remains poorly understood. By integrating genetic, epigenetic, and functional analyses at the single cell level, we show that progression of uveal melanoma (UM), the most common intraocular primary cancer in adults, is driven by loss of Polycomb Repressive Complex 1 (PRC1) in a subpopulation of tumor cells. This leads to transcriptional de-repression of PRC1-target genes and mitotic chromosome segregation errors. Ensuing CIN leads to the formation of rupture-prone micronuclei, exposing genomic double-stranded DNA (dsDNA) to the cytosol. This provokes tumor cell-intrinsic inflammatory signaling, mediated by aberrant activation of the cGAS-STING pathway. PRC1 inhibition promotes nuclear enlargement, induces a transcriptional response that is associated with significantly worse patient survival and clinical outcomes, and enhances migration that is rescued upon pharmacologic inhibition of CIN or STING. Thus, deregulation of PRC1 can promote tumor progression by inducing CIN and represents an opportunity for early therapeutic intervention.

## Abstract (web summary)

The molecular underpinnings driving uveal melanoma (UM) progression are unknown. Here the authors show that loss of Polycomb Repressive Complex 1 triggers chromosomal instability, which promotes inflammatory signaling and migration in UM.

## Introduction

**Fig. 1:**
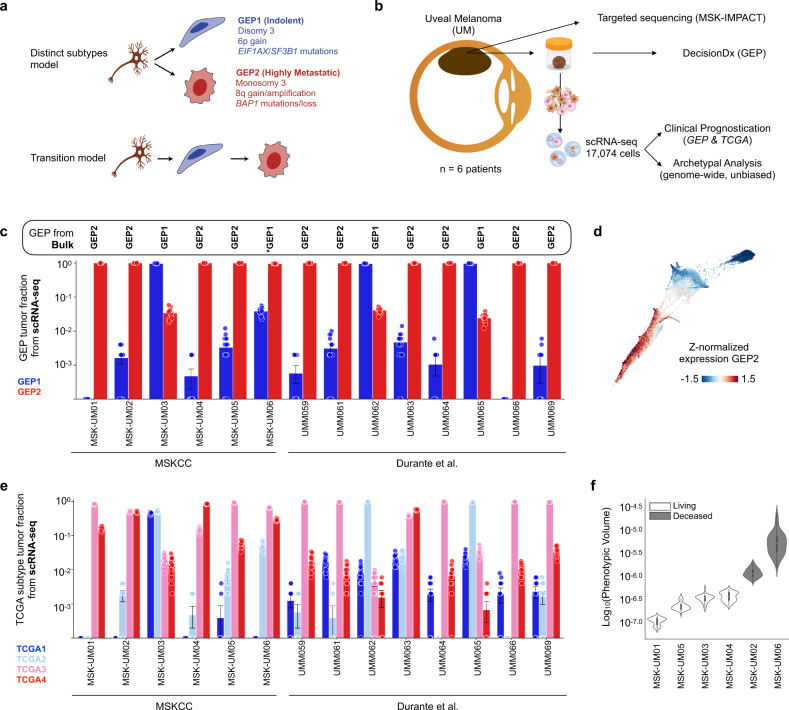
Phenotypic continuum of disease progression in primary UM. **a** Distinguishing features of uveal melanoma (UM) with good (blue) and poor (red) prognosis; highlighting two potential models of disease progression. GEP gene expression profile, TCGA the Cancer Genome Atlas. **b** Patient tissue profiling (metadata summarized in Supplementary Table 1). Immediately following enucleation, six primary tumor specimens were obtained for clinical prognostic and single cell transcriptional profiling. Targeted sequencing using the MSK-IMPACT platform was performed on the formalin-fixed, paraffin embedded enucleation specimens. **c** Bulk GEP classification assigned to each patient according to the DecisionDx test (Castle Biosciences) (box). Individual tumor cells were likewise assigned to GEP1 (blue) vs. GEP2 (red) clinical prognostic groups according to their average expression of the GEP prognostic gene signatures using a two-component Bayesian Gaussian Mixture Model (BGMM, “Methods”). The fraction of individual tumor cells assigned to GEP1 (blue) and GEP2 (red) per patient is visualized in the bar graphs, where bootstrapping was used to correct for number of cells per patient (bar, mean; whiskers, 95% confidence intervals, 500 tumor cells sampled over n = 20 random subsets of the data). Asterisks, highlight a patient in which there was a discrepancy between DecisionDx bulk classification and the majority GEP classification prescribed by our single cell analysis of resected tumors. Notably, this patient (MSK-UM06) experienced metastatic progression and succumbed to their disease within six months of diagnosis (Supplementary Table 1). Intra-tumoral prognostic heterogeneity was validated in a second, independent cohort recently published by Durante et al.^24^. **d** Force-directed layout of all patient tumor cells colored by z-normalized imputed expression of the average GEP2 gene signature. **e** Individual tumor cells were likewise assigned to one of the four TCGA molecular subtypes of UM according to their average expression of characteristic genes using a four-component BGMM (Methods) in our cohort and a second, independent cohort recently published by Durante et al.^24^. The fraction of individual tumor cells assigned to TCGA subtype 1 (dark blue), subtype 2 (light blue), subtype 3 (pink) and subtype 4 (red) per patient is visualized in the bar graphs, where bootstrapping was used to correct for number of cells per patient (bar, mean; whiskers, 95% confidence intervals, 500 tumor cells sampled over *n* = 20 random subsets of the data). **f** Violin plots showing the distribution of intra-patient phenotypic volume (defined as the pseudo-determinant of the gene expression covariance matrix, detailed in “Methods”), controlled for number of cells and labeled by patient status (alive vs. deceased). Distributions represent 100 random subsamples from the data (*n* = 150 cells per patient). Overlaid bar and whisker plots reflect the mean and interquartile range.

Uveal Melanoma (UM), a lethal eye cancer of adults and the second most common subtype of melanoma, is characterized by striking variability in metastatic tendency^1–3^. Once metastases are detected, median survival is less than twelve months^4–9^. Therefore, identifying patients at high-risk for metastasis and developing ways to intervene are critical priorities. Highly metastatic UM tumors differ from their more indolent counterparts in at least three ways^1,3^. First, they tend to have an “epithelioid” morphology with enlarged nuclei^10,11^. Second, they are often monosomic for chromosome 3^2,12,13^, and frequently harbor mutations in the *BAP1* gene (located on chromosome 3)^2,14^—a component of the polycomb repressive deubiquitinase (PR-DUB) complex that hydrolyzes ubiquitin at lysine 119 of the repressive Histone 2A (H2AK119)^15,16^. Third, they exhibit a distinctive gene expression signature^2,17^. A clinically-validated 12-gene signature—representative of the transcriptional changes that distinguish the two prognostic groups—is often used to identify patients with low-risk UM (Gene expression profile 1, GEP1) and high-risk tumors (Gene expression profile 2, GEP2)^18,19^ in the clinical setting. Currently, it is not known whether high-risk and low-risk UMs are fundamentally distinct disease subtypes or whether genetic and/or epigenetic changes in a subpopulation of tumors cells can lead to evolution from a relatively indolent to an aggressive UM (Fig. 1a). Identifying the underlying molecular basis for such a transition would provide insight into the molecular pathogenesis of UM and yield a critical opportunity for early therapeutic intervention. Here, we demonstrate that UM progression is driven by loss of PRC1 in a subpopulation of tumor cells, leading to transcriptional de-repression of PRC1-target genes and mitotic chromosome segregation errors. Hence, tumor stratification based on bulk transcriptional profiling of an inherently heterogeneous tumor is likely biased by detection of the most common tumor cell subpopulation.

## Results

### Single cell transcriptional landscape of uveal melanoma tumors

To resolve intra-tumoral heterogeneity and gain insights into tumor evolution that cannot be resolved by bulk tumor profiling^20,21^, we profiled the transcriptomes of 17,074 individual cells obtained from six freshly enucleated UM specimens from different prognostic categories (Fig. 1b). Immediately after enucleation, tumor specimens were obtained and sent for GEP clinical prognostication testing (DecisionDx) to assign individual patients to GEP prognostic classes^19^. According to DecisionDx, four of the six tumors were classified as high-risk GEP2 whereas the remaining two tumors were classified as low-risk GEP1 (Supplementary Table 1). Formalin-fixed, paraffin embedded specimens were also sent for targeted exome sequencing using the MSK-IMPACT platform^22^ (Fig. 1b, Supplementary Table 1). Fresh tumor samples analyzed with single-cell RNA sequencing (scRNA-seq) were derived from enucleation specimens without enrichment for a specific cell type, such that a piece of the viable tumor and its microenvironment were sampled in an unbiased manner. The library size, complexity, and viability metrics were of high quality (Supplementary Fig. 1a–c) and largely consistent across patients (Supplementary Fig. 1d, e). All scRNA-seq data were merged and normalized to create a global cell atlas, clustering^23^ of which revealed 27 cell types and states spanning retinal, immune and cancer cells (Supplementary Fig. 1f, g). Retinal and immune cell types were highly reproducible across patients, whereas patient-specific cell states were observed within tumor cell populations (Supplementary Fig. 1h–j). The majority of cells analyzed were tumor cells, expressing genes consistent with a melanocytic cell of origin (Supplementary Fig. 1i) and showing significant chromosome copy number alterations, including those canonically associated with UM (Supplementary Fig. 2a, b). In addition, we obtained single cell RNA sequencing data from an independent cohort of eight primary UM to validate key findings^24^.

### Intratumoral phenotypic heterogeneity in UM tumors

Individual tumor cells were assigned to a prognostic class based on their average imputed expression of GEP1 and GEP2 discriminate genes using a two-component Bayesian Gaussian Mixture Model (Supplementary Fig. 3a, b, Methods). Strikingly, five tumors contained a heterogeneous admixture of cells that resembled both prognostic classes to varying extent (Fig. 1c, *MSKCC*). Likewise, the same classifier applied to the independent cohort of eight primary UM^24^, also revealed that the majority of primary tumors (7 out of 8) contain a heterogeneous admixture of tumor cells that resemble both prognostic classes to varying degrees, even when controlling for sampling differences across patients (Fig. 1c, Durante et al.^24^). Rather than observing two distinct states, we detected a continuum from a GEP1-like to a GEP2-like phenotype at the level of individual cells (Fig. 1d). Consistent with the previously described relationship between *BAP1* loss and aggressive phenotype^2,3,14,25–31^, the GEP1-to-GEP2 transition was highly correlated with reduced *BAP1* expression (Supplementary Fig. 3c) and the monosomy 3-associated gene expression signature^17^ (Supplementary Fig. 3d). Notably, GEP classification based on bulk transcriptional profiling (DecisionDx) was discordant with the majority GEP class detected by scRNA-seq in one out of six cases in the MSK dataset (Fig. 1c and Supplementary Table 1). A tumor (MSK-UM06) was classified as GEP1 based on bulk transcriptional sampling, yet harbored predominantly GEP2 cells (96.1%) by single cell analysis. This patient experienced metastatic progression and succumbed to disease within 6 months of diagnosis, in line with an abundance of aggressive tumor cells and highlighting the limitations of tumor stratification based on bulk transcriptional profiling of an inherently heterogeneous tumor.

While GEP classification is widely used clinically for diagnostic purposes, it has both technical and biological limitations. Detailed analysis of the TCGA uveal melanoma cohort (integrative analysis of UM transcriptomes, methylomes and genomic copy number data, *n* = 80 patients) has revealed the existence of four molecularly distinct biological and prognostic subsets of UM^2^. To likewise assess intra-tumor heterogeneity in the context of these molecularly distinct subsets, a four-component Bayesian Gaussian Mixture Model was applied to probabilistically assign individual tumor cells to each subtype based on their average imputed expression of characteristic genes (Fig. 1e and Supplementary Fig. 3e, f, Methods). Individual tumor cells promiscuously expressed markers associated with multiple TCGA subtypes (Supplementary Fig. 3g) and nearly all tumors across both cohorts^24^ showed substantial intra-tumoral heterogeneity when tumor cells were assigned to their maximally probable subtype (Fig. 1e). Therefore, and regardless of how risk levels are defined, individual UM tumors contained a heterogeneous admixture of cells that resembled different molecular subtypes to varying degrees. To capture this intra-patient cell state complexity—independent of clinical gene expression signatures—we applied a phenotypic volume metric^32^ across all variably expressed genes expressed by tumor cells within each patient. Notably, the two patients exhibiting the highest levels intra-tumoral phenotypic complexity succumbed to metastatic disease during the course of this study (Fig. 1f). Such intratumor heterogeneity - which cannot be resolved by bulk sequencing—suggests a model of UM progression; whereby cells within the primary tumor exist along various stages of an evolutionary continuum from an indolent towards a more aggressive phenotype.

The unexpected phenotypic progression underlying this cell state diversity (Fig. 1d) motivated us to apply archetypal analysis^33^ for unbiased, genome-wide transcriptomic characterization of tumor phenotypic states^34^. Analysis revealed 8 tumor cell archetypes, which are labeled on the force directed layout in Supplementary Fig. 4a. When individual tumor cells were assigned to their nearest archetype (“Methods”), each patient showed accumulation of multiple phenotypic states defining disease progression (Supplementary Fig. 4b). A local neighborhood of cells around each archetype was used to characterize genes differentially expressed in these bounding phenotypic states (Supplementary Fig. 4c). Archetypes were distinguished by differential expression of key pathways related to inflammatory response programs, aneuploidy, chromatin modifications and UM prognostic classifications. (Supplementary Fig. 4d). Collectively, this suggests such processes may underlie the evolution of UM tumors from an indolent to an aggressive phenotype.

### Loss of PRC1 activity defines high-risk UM

**Fig. 2:**
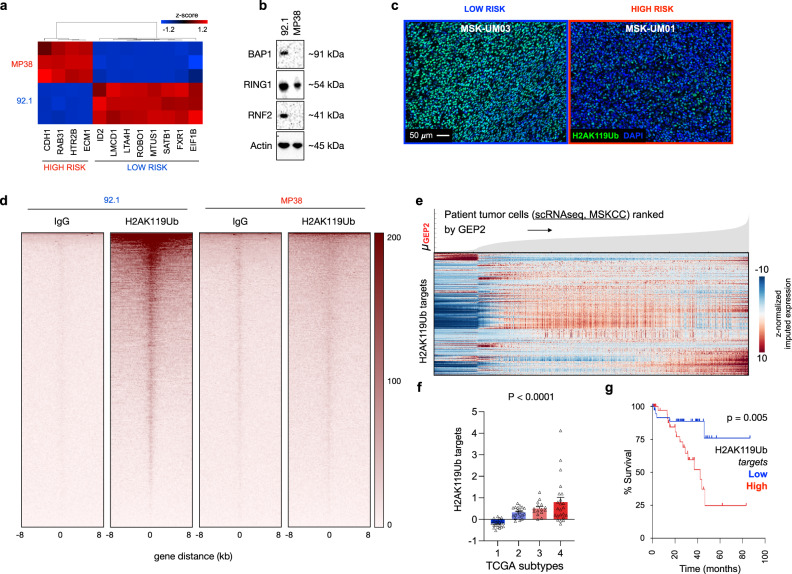
Loss of PRC1-mediated transcriptional repression in high-risk UM. **a** Unbiased hierarchical clustering of UM cell lines, 92.1 and MP38, in biological triplicates, based on normalized FPKM values of the GEP 12-discriminant geneset^19^. **b** Western blot of BAP1 and PRC1 core ligases RING1 and RNF2 relative to actin in 92.1 and MP38 cells. Data representative of biological triplicates. **c** Immunofluorescence of ubiquitinated H2A (green) and DAPI (blue) in MSK-UM03 (greatest proportion of GEP1 tumor cells in the cohort) and MSK-UM01 (greatest proportion of GEP2 tumor cells in the cohort). Representative images from six biological specimens. **d** Heatmaps representing CUT&RUN intensities of H2AK119Ub normalized to IgG in 92.1 and MP38. Data representative of biological duplicates. **e** Expression of H2AK119Ub target genes for individual tumor cells ranked by average imputed expression of the GEP2 gene signature (gene signatures annotated in Supplementary Data File 1) in ascending order from left to right. For each gene, imputed expression was z-normalized across all cells and smoothed using a 20-cell moving average window. Top, filled area plot showing average expression of GEP2 signature genes across ranked tumor cells. **f** Expression of H2AK119Ub target genes across the 4 molecular TCGA subtypes. Statistical significance tested using one-way ANOVA; *p* = 7.3 × 10^−6^; *n* = 80. Bars, mean of average expression; error bars, standard error of the mean. **g** Overall survival of (*n* = 80) TCGA-UM patients with primary tumors stratified by high (top 50th percentile, *n* = 40) and low (bottom 50th percentile, *n* = 40) expression of the ‘H2AK119Ub targets’ geneset. Statistical significance tested using two-sided log-rank test.

To better understand the molecular underpinnings of UM single cell heterogeneity and tumor progression, we sought experimental models that recapitulate its distinct biological and prognostic classes. Towards this, we performed RNA sequencing of five established UM cell lines from diverse genetic backgrounds^35–37^. Cell lines distinctly clustered based on expression of the 80 characteristic genes that define the four major TCGA-UM subtypes or the 12-gene module that defines the GEP prognostic groups^19^ **(**Supplementary Fig. 5a–c**)**. We designated cell lines falling at the opposite end of the gene expression spectrum, 92.1 and MP38, as low-risk and high-risk UM cells, respectively (Fig. 2a and Supplementary Fig. 5a). Expectedly, high-risk UM cells, MP38, harbored *BAP1* mutation^35^, and had no detectable BAP1 protein (Fig. 2b). This is in line with reports showing that *BAP1* genomic loss is a defining feature of aggressive UM^1–3,14^.

BAP1 hydrolyzes monoubiquitin on H2AK119Ub, a transcriptional repressive histone posttranslational modification (PTM)^15,16,38,39^. To test whether BAP1 loss translated into reduced H2AK119Ub, we performed H2AK119Ub immunofluorescence. To our surprise, high risk UM cells exhibited lower H2AK119Ub compared to their low risk counterparts (Supplementary Fig. 6a, b). Genome-wide localization analysis for H2AK119Ub, using Cleavage Under Targets and Release Using Nuclease (CUT&RUN)^40^, was consistent with the data from immunostaining. High-risk UM cells, MP38, exhibited near-complete loss of H2AK119Ub deposition at target loci compared to their low-risk counterparts, 92.1 (Fig. 2d). We next validated this finding in patient samples and found that the patient with predominantly low-risk features (e.g., MSK-UM03) exhibited significantly higher H2AK119Ub staining compared to those with high-risk gene expression profiles (e.g., MSK-UM01) (Fig. 2c).

Ubiquitiylation of H2AK119 is mediated by PRC1 through its ubiquitin ligase activity^15,41^. PRC1 contains a conserved core which consists of a RING1 (Really Interesting New Gene 1) or RNF2, both of which possess E3 ubiquitin ligase activity, in addition to one of the six PcG ring-finger domain proteins (PCGF1-6)^15^. PRC1 core ligase activity is necessary to achieve transcriptional repression of target genes^41,42^. Consistent with a reduction in H2AK119Ub in high risk UMs, transcript and protein levels of the core PRC1 ligases, RING1 and RNF2, were indeed lower in MP38 high-risk UM cells (Supplementary Fig. 6c and Fig. 2b). Similarly, in the TCGA cohort, UM tumors with *BAP1* loss exhibited significantly lower *RING1* and *RNF2* expression levels compared to tumors with intact *BAP1*, respectively (Supplementary Fig. 6d, e). Of all PRC1 accessory components (PCGF1-6), four (PCGF2,3,6 and PCGF4, also known as BMI1) exhibited reduced expression in MP38 cells compared to 92.1 (Supplementary Fig. 6f). In addition, expression of *JARID2*, a PRC2 accessory protein that is distinguished by its binding affinity to H2AK119Ub^43,44^, was also reduced in both MP38 cells compared to 92.1, as well as in human tumors with *BAP1* loss (Supplementary Fig. 6g, h). Similar to H2AK119Ub, genome-wide localization analysis demonstrated near-loss of PRC1 components, RING1, RNF2, and BMI1 deposition in MP38, compared to 92.1 (Supplementary Fig. 7a). On the contrary, genomic loci bound to trimethylated Histone 3 at Lysine 27 (H3K27me3), another transcriptional repressive histone PTM were similar between 92.1 and MP38 (Supplementary Fig. 7a). Collectively this suggests widespread reduction of PRC1 components in high-risk UM.

In line with these findings, elevated expression of either *JARID2* or *RING1* mRNA was significantly associated with prolonged overall survival and decreased risk of metastasis (Supplementary Fig. 7b, c). Both *JARID2* and *RING1* are located on chromosome 6p, in line with prior observations that in UM, 6p gain is associated with a good prognosis^2,45,46^. We then asked whether low *JARID2* and *RING1* levels confer poor prognosis independent of 6p gain. The association between either JARID2 or RING1 and UM metastasis or death was assessed using logistic regression where 6p gain was a co-variate. *JARID2* levels were inversely correlated with survival (Odds ratio (OR), 0.50; 95% confidence interval (CI), 0.31–0.74), and metastasis (OR, 0.69; CI, 0.50–0.91). Similarly, *RING1* levels were inversely correlated with survival (OR, 0.61; CI, 0.37–0.89) and metastasis (OR, 0.65; CI, 0.43–0.89), indicating that higher expression levels of *JARID2* or *RING1* confer good prognosis independent of 6p gain.

We then asked whether a loss of PRC1 and H2AK119Ub translates into loss of transcriptional repression of target genes. We curated a gene list from genomic peaks of H2AK119Ub in UM cells (*H2AK119Ub targets*). These genes were highly expressed in patient tumor cells with increasing GEP2-like features (Fig. 2e and Supplementary Fig. 7d) (MSKCC and Durante et al.^24^), exhibited progressive de-repression across the 4 molecular TCGA subtypes (Fig. 2f), and foretold poor prognosis (Fig. 2g, TCGA). We interrogated the expression levels of genes that are bound to H3K27me3, excluding H2AK119Ub targets, and found that their levels were similar across the 4 molecular TCGA subtypes and did not correlate with overall survival (Supplementary Fig. 7e, f). Collectively, these results indicate that loss of H2AK119Ub and PRC1-mediated transcriptional repression is a feature of high-risk UM—despite concurrent loss of *BAP1*.

### PRC1 inhibition phenocopies UM progression

**Fig. 3:**
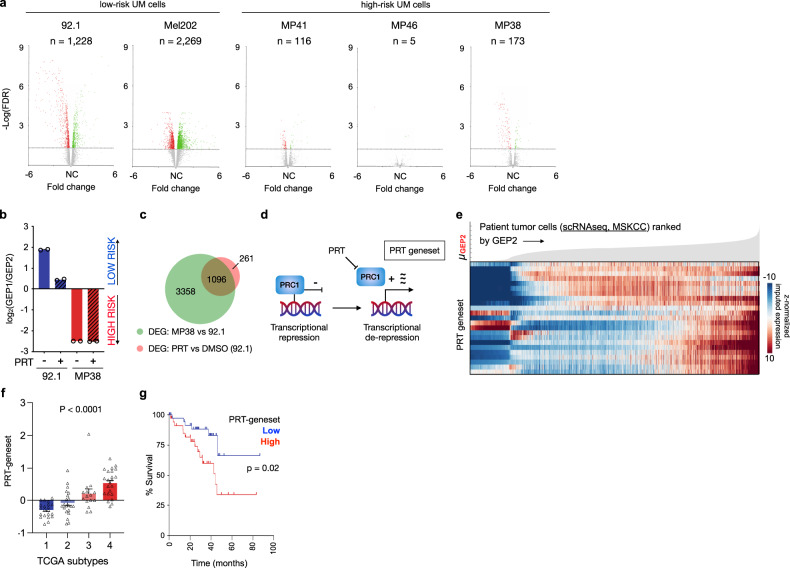
Evolution of aggressive UM phenotype triggered by PRC1 inhibition. **a** Volcano plots showing differentially expressed genes upon PRT-treatment across five UM cell lines ranked according to their GEP1/GEP2 score (see Supplementary Fig. 5a–c). Fold change for individual genes is shown as a function of significance, -log_10_(FDR). Genes with FDR value less than 0.05 and fold change greater or less than 1, were highlighted in green and red, respectively. Genes not meeting significance (FDR > 0.05) are shown as gray. Number of genes meeting significance (FDR < 0.05) are annotated per each cell line. **b** Ratio of GEP1/GEP2 average gene signature expression (gene signatures annotated in Supplementary Data File 1) in low-risk UM cells (92.1) and high-risk UM cells (MP38) upon 24 h DMSO or PRT-treatment; FPKM values obtained from bulk RNA-seq are reported (bar, mean; circles, biological duplicates). **c** Venn diagram of differentially expressed genes (DEG) upon PRT treatment (red) and between high-risk UM cells (MP38) and low-risk UM cells (92.1) (green). **d** A schematic showing transcriptional de-repression upon pharmacologic inhibition of PRC1 using PRT (“PRT-geneset” annotated in Supplementary Data File 1). **e** Expression of the PRT-geneset across individual tumor cells ranked by average imputed expression of the GEP2 gene signature (gene signatures annotated in Supplementary Data File 1) in ascending order from left to right. For each gene, imputed expression was z-normalized across all cells and smoothed using a 20-cell moving average window. Top, filled area plot showing average expression of the GEP2 signature across ranked tumor cells. **f** Average expression of genes upregulated upon PRT-treatment of 92.1 cells (‘PRT-geneset’) across the 4 molecular TCGA subtypes. Statistical significance tested using one-way ANOVA; *p* = 9.6 × 10^−9^; *n* = 80. Bars, mean of average expression; error bars, standard error of the mean. **g** Overall survival of (*n* = 80) TCGA-UM patients with primary tumors stratified by high (top 50th percentile, *n* = 40) and low (bottom 50th percentile, *n* = 40) average expression of the “PRT-geneset”. Statistical significance tested using two-sided log-rank test.

To determine if loss of PRC1-mediated transcriptional repression underlies the transition from low-risk to high-risk UM, we used PRT4165 (thereafter referred to as PRT), a specific inhibitor of RING1 and RNF2 ligase activity, which is necessary to maintain PRC1-mediated target gene repression^41,42^. PRT has been shown to abolish ubiquitylated H2AK119 within 1 h of treatment^47^. PRT-treatment of low-risk 92.1 UM cells reduced H2AK119Ub levels after 2 h of treatment to those seen in MP38 cells (Supplementary Fig. 8a–d). On the other hand, treatment of high-risk MP38 UM cells, which have already adapted low basal activity of PRC1, had no impact on H2AK119Ub levels (Supplementary Fig. 8a, b). Importantly, PRC1 inhibition led to a profound transcriptional change in low-risk UM cells (92.1 and Mel202) while having minimal impact on gene expression in high-risk UM cells (MP41, MP46, and MP38) (Fig. 3a). Furthermore, treatment with PRT resulted in transcriptional upregulation of GEP2 signature genes and downregulation of GEP1 signature genes after just 24 h of treatment, in low-risk—but not high-risk—UM cells (Fig. 3b). On the contrary, inhibition of the H3K27 methyltransferase, EZH2, using the clinical grade inhibitor, EPZ, did not lead to transcriptional alterations in GEP1/2 signatures (Supplementary Fig. 8e). The majority (80.8%) of genes differentially expressed upon PRT treatment in low-risk UM cells were also differentially expressed between high-risk and low-risk UM cells at baseline (Fig. 3c).

Concordantly, genes upregulated upon pharmacologic inhibition of PRC1 in low-risk UM cells (fold change > 1 and Bonferroni <0.05, “*PRT-geneset*” defined in Fig. 3d) were highly expressed in patient tumor cells with increasing GEP2-like features (Fig. 3e and Supplementary Fig. 8f). These genes exhibited progressive de-repression across the 4 molecular TCGA subtypes (Fig. 3f) and foretold poor prognosis (Fig. 3g, TCGA), underscoring that loss of PRC1-mediated transcriptional repression is a feature of high-risk UM. Two of the 4 genes that define the GEP2 profile, *ECM1* and *HTR2B*, were among the most differentially expressed genes in the PRT-geneset (*n* = 28) (Supplementary Data File 1).

**Fig. 4:**
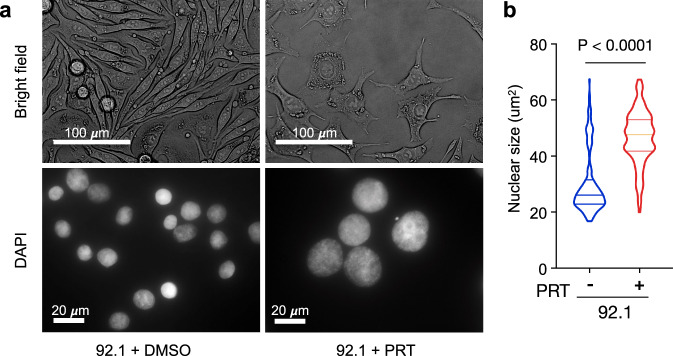
Morphological changes and nuclear enlargement induced by PRC1 inhibition. **a** Top panel, bright field light microscopy of low-risk UM cells (92.1) upon long-term DMSO and PRT-treatment. Bottom panel, DAPI staining of low-risk UM cells (92.1) after 48 h of PRT, and DMSO treatment. Images representative of experimental triplicates. **b** Violin plots showing the distribution of nuclei size in low-risk UM cells (92.1) upon PRT (*n* = 152) and DMSO (*n* = 163) treatment for 48 h. Lines distinguish interquartile ranges; width reflects observed nuclei number. Statistical significance tested using two-sided unpaired Student’s *t* test, *p* = 4.9 e^−36^. Source data are provided as a Source Data file.

In addition to transcriptional changes, PRT treatment led to profound morphologic alterations that have long been associated with high-risk UM tumors^10,11^; mainly enlarged nuclei and an epithelioid morphology (Fig. 4a, b). In line with the drug’s specificity to low-risk UM cells, characterized by elevated PRC1 activity, treatment of high-risk, PRC1-defecient MP38 cells with PRT had no effects on nuclear size and morphology (Supplementary Fig. 8g). Similarly, PRC1 inhibition also impaired growth of low-risk 92.1 cells, but not high-risk MP38 cells (Supplementary Fig. 8h). Notably, 92.1 cells treated with PRT grew at a slower rate, similar to high-risk UM cells, however reduced growth rates of PRT-treated 92.1 cells quickly rebounded to baseline following PRT withdrawal (Supplementary Fig. 8i). Collectively, these results suggest that loss of PRC1 activity recapitulates a transition from low-risk to high-risk UM—linking critical genetic, epigenetic, and cytologic features.

### Widespread CIN and inflammatory signaling in high-risk UM

**Fig. 5:**
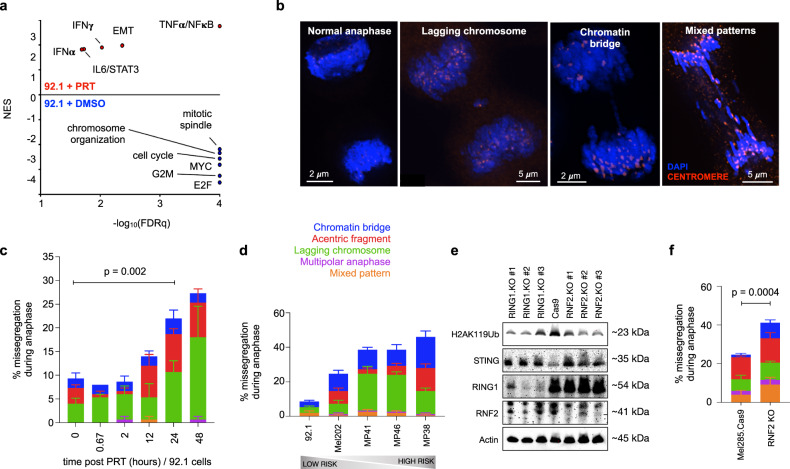
Increased chromosome segregation errors upon PRC1 inhibition. **a** Volcano plots showing the top differentially expressed pathways in low-risk UM cells (92.1) treated with PRT vs DMSO for 24 h; evaluated across biological triplicates. Normalized enrichment score (NES) for selected genesets shown as a function of significance, -log_10_(FDRq); FDRq, Bonferroni corrected *p* value. An unfiltered list of all significant gene signatures is provided in Supplementary Data File 5. **b** Examples of UM cells in anaphase stained for DAPI (blue) and centromeres (red); demonstrating different patterns of chromosome segregation errors. Additional patterns of missegregation are shown in Supplementary Fig. 9b. Representative images from biological triplicates. **c** Abundance of chromosome segregation error patterns during anaphase in low-risk UM cells (92.1) upon PRT-treatment as a function of time. Statistical significance tested using two-sided unpaired Student’s *t* test. Source data are provided as a Source Data file. **d** Abundance of chromosome segregation error patterns during anaphase across UM cell lines, arranged from left to right based on their GEP2 score. Source data are provided as a Source Data file. **e** Western blot of H2AK119Ub, STING, RING1, and RNF2 relative to Actin in Mel285 cells upon RING1 and RNF2 knockout. Data representative of biological triplicates. **f** Abundance of chromosome segregation error patterns during anaphase in UM cells (Mel285.Cas9) upon RNF2 knockout. Statistical significance tested using two-sided unpaired Student’s *t* test. Source data are provided as a Source Data file. Stacked bars, mean of each missegregation pattern; error bars, standard error of the mean across experimental triplicates (**c–f**).

To further explore the molecular underpinning of this transition, we performed gene set enrichment analysis (GSEA) comparing DMSO and PRT-treated low-risk UM cells. In addition to PRC1 target genes, PRT treatment led to significant upregulation of pathways related to inflammation, such as NF-κB (Normalized enrichment score (NES), 2.8; FDRq <0.001), IL6/STAT3 (NES, 1.8; FDRq, 0.02) and epithelial-to-mesenchymal transition (EMT) (NES, 2.0; FDRq, 0.04). Conversely, there was downregulation of pathways involved in the cell cycle and mitotic spindle assembly (Fig. 5a). Differentially expressed pathways upon PRT treatment were reminiscent of those seen enriched in high-risk MP38 as compared to low-risk 92.1 UM cells, with the latter exhibiting upregulation of inflammation and EMT-related gene sets (Supplementary Fig. 9a).

Given increased aneuploidy in high-risk primary UM^2^ and increased inflammatory signaling (Fig. 5a) we observed in aggressive UM, as well as in other studies^2,25,26,48–52^, we set out to determine whether changes in PRC1 could potentially alter genomic stability. We have recently shown that errors in chromosome segregation—a defining feature of cancer cells with chromosomal instability (CIN)—can generate micronuclei, which, upon rupture, expose their enclosed genomic double-stranded DNA (dsDNA) to the cytosol^53–56^. This leads to aberrant activation of the cGAS-STING cytosolic dsDNA signaling pathway and downstream inflammatory signaling mediated by noncanonical NF-κB activation, as well as upregulation of EMT and migratory pathways that promote metastatic progression^56^. We hypothesized that loss of PRC1 function may trigger CIN, leading to enhanced migration and inflammatory signaling characteristic of high-risk UM. Indeed, PRT-treatment of low-risk 92.1 cells led to increased frequency of mitotic chromosome segregation errors, as evidenced by the preponderance of lagging chromosomes and acentric chromatin fragments in PRT-treated cells (Fig. 5b, c and Supplementary Fig. 9b). Importantly these defects emerged 24–48 h after drug treatment (Fig. 5c) arguing against a direct, short-term effect on the mitotic spindle and instead favoring a transcriptional response. Importantly, and in line with our earlier observations demonstrating PRT’s specificity to UM cells with intact PRC1, PRT treatment of high-risk MP38 cells showed no effect on chromosome segregation errors (Supplementary Fig. 9c). Correspondingly, intrinsic rates of chromosome missegregation correlated with the ratio of GEP2/GEP1 gene expression (Fig. 5d). Unlike PRC1, Inhibition of EZH2—the core catalytic component of the PRC2 complex—did not lead to an increase in chromosome segregation defects (Supplementary Fig. 9d). We then complemented these pharmacologic modulations with genetic manipulation of core PRC1 ligases. First, we established a CRISPR-Cas9 knockout system in Mel285, a low-risk uveal melanoma cell line which expresses BAP1^57^. Knockout of either RING1 or RNF2 resulted in a significant reduction in H2AK119Ub levels (Fig. 5e), and loss of core PRC1 components dramatically increased chromosome segregation defects observed during anaphase (Fig. 5f).

**Fig. 6:**
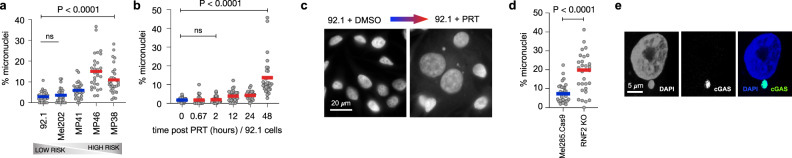
Cytosolic DNA exposure in high-risk UM. **a** Baseline micronuclei frequency across UM cell lines, arranged from left to right based on their GEP2 score. Bar represents median; points, measured frequency of micronuclei per high-power field evaluated across three experimental replicates. Statistical significance tested using two-sided student *t* test; *p* = 2.8 × 10^−8^. Source data are provided as a Source Data file. **b** Micronuclei frequency in low-risk UM cells (92.1) upon PRT treatment as a function of time. For (**a**, **b**), bar represents median; points, measured frequency of micronuclei per high-power field evaluated across three experimental replicates. Statistical significance tested using two-sided student *t* test; *p* = 7.7 × 10^−8^. Source data are provided as a Source Data file. **c** Low-risk UM cells (92.1) stained for DAPI (white); showing increased nuclear size and micronuclei formation upon PRT-treatment. Images representative of experimental triplicates. **d** Micronuclei frequency in UM cells (Mel285.Cas9) upon RNF2 knockout. Bar represents median; points, measured frequency of micronuclei per high-power field evaluated across three experimental replicates. Statistical significance tested using two-sided student *t* test; *p* = 7.7 × 10^−6^. Source data are provided as a Source Data file. **e** An example of a cGAS (green) localization to micronuclei (blue). Representative image from biological triplicates.

To test whether increased chromosome missegregation during mitosis leads to the formation of micronuclei, we assessed the frequency of micronuclei in the various UM cell lines and found rates of micronuclei mirrored those of chromosome missegregation. High-risk UM cells had significantly higher rates of micronuclei compared to their low-risk counterparts (Fig. 6a). Similarly, PRT treatment of 92.1 cells—but not of high-risk MP38 cells—led to a significant increase in micronuclei (Fig. 6b, c and Supplementary Fig. 9e), whereas treatment with an EZH2 inhibitors had no impact on their formation (Supplementary Fig. 9f). Likewise, genetic knockout of RNF2 resulted in a significant increase in micronuclei frequency (Fig. 6d). Importantly, these micronuclei frequently co-localized with cGAS (Fig. 6e), indicative of cytosolic exposure of their enclosed genomic dsDNA and activation of cytosolic dsDNA sensing, cGAS-STING pathway.

**Fig. 7:**
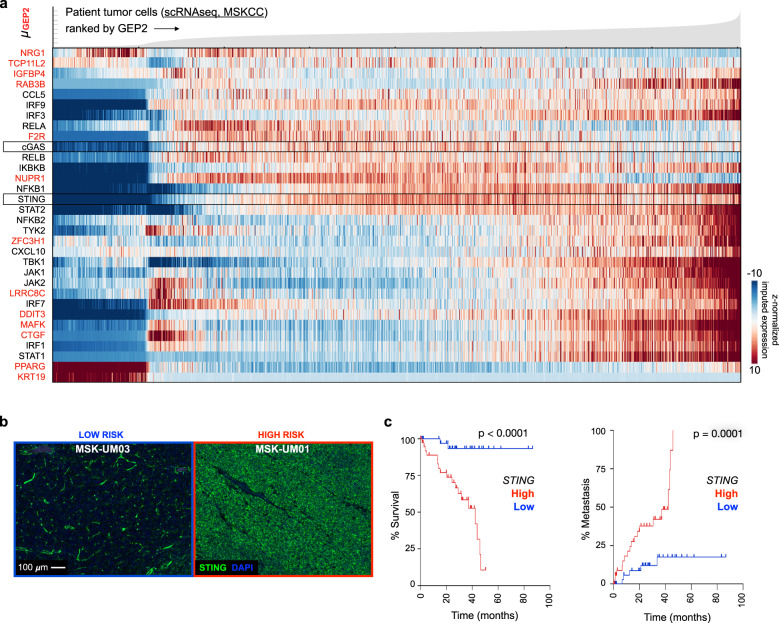
Cell-intrinsic inflammation in high-risk UM. **a** Expression of key cytosolic nucleic acids sensors, intermediate signaling adapters, executioners, interferon stimulated genes (ISGs) and CIN-induced non-canonical NF-kB targets (shown in red)^56^ for all patient tumor cells ranked by average imputed expression of the GEP2 gene signature (gene signatures annotated in Supplementary Data File 1) in ascending order from left to right; genes are clustered using an Euclidean distance metric. For each gene, imputed expression was z-normalized across all cells and smoothed using a 20-cell moving average window. Top, filled area plot showing average expression of the GEP2 signature across ranked tumor cells. **b** Immunofluorescence of STING (green) and DAPI (blue) in MSK-UM03 (greatest proportion of GEP1 tumor cells in the cohort) and MSK-UM01 (greatest proportion of GEP2 tumor cells in the cohort). Representative images from six biological specimens. **c** Overall survival (left) and UM-related metastasis (right) of (*n* = 80) TCGA-UM patients with primary tumors stratified by expression of high (top 50th percentile, *n* = 40) and low (bottom 50th percentile, *n* = 40) *STING*. Statistical significance tested using two-sided log-rank test; *p* (left) = 2.2 × 10^−5^.

In line with these findings, genetic knockout of either RING1 or RNF2 resulted in increased STING levels (Fig. 5e). We also observed upregulation of *CGAS*, STING *(TMEM173)* mRNA and downstream inflammatory response pathway effectors induced by CIN, including noncanonical NF-κB targets in patient tumor cells ranked according to the average GEP2 gene expression signature (Fig. 7a). These target genes are upregulated in response to chronic STING activation in cancer cells with CIN^56^. We validated these findings in an independent cohort^24^, where we likewise observed upregulation of *CGAS, TMEM173* and downstream inflammation-related mRNAs, including non-canonical NF-κB targets, in high-risk tumor cells (Supplementary Fig. 10a). Concordantly, *STING* and *cGAS* mRNA expression levels were elevated in TCGA-UM tumors with genomic copy number loss in *BAP1* compared to BAP1-intact tumors (Supplementary Fig. 10b). To further validate these findings in human tumor samples, we performed immunofluorescence imaging of STING and found elevated expression of tumor cell-intrinsic STING in the predominantly high-risk tumor (e.g., MSK-UM01) as compared to the predominantly low-risk tumor (e.g., MSK-UM03), where minimal STING expression was mainly restricted to the stromal compartment (Fig. 7b). In the TCGA cohort, *TMEM173* expression was highly prognostic, whereby high STING levels predicted metastasis and reduced overall survival (Fig. 7c, TCGA). All together, these results suggest that CIN-induced cytosolic dsDNA signaling downstream of PRC1 loss, is a feature of high-risk UM.

### PRC1 inhibition promotes CIN and STING-dependent migration

**Fig. 8:**
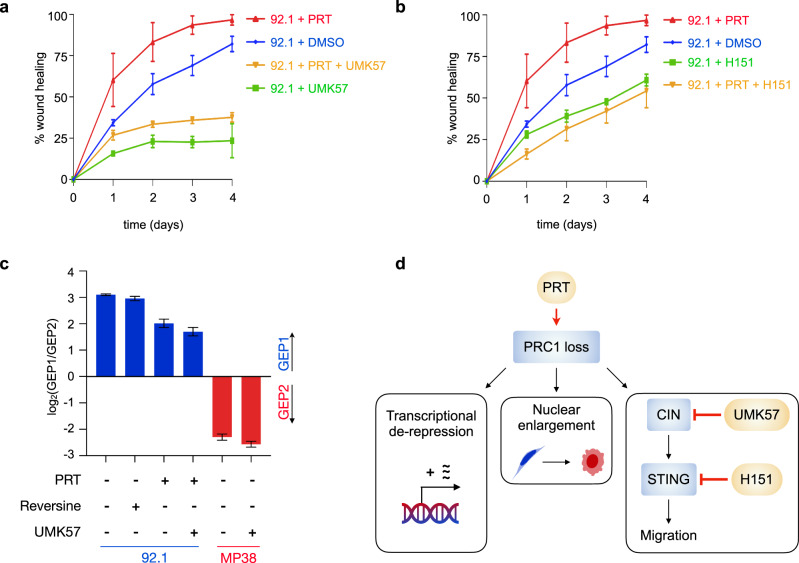
PRC1 inhibition promotes CIN and STING-dependent migration. **a** Wound scratch assay to assess migratory potential of low-risk UM cells (92.1) upon treatment with DMSO, PRT, UMK57 (CIN inhibition) or PRT and UMK57. Data obtained from three experimental replicates. Data points, mean; error bars, standard error of the mean. Source data are provided as a Source Data file. **b** Wound scratch assay to assess migratory potential of low-risk UM cells (92.1) upon treatment with DMSO, PRT, H151 (STING inhibition) or PRT and H151. Data obtained from three experimental replicates. Data points, mean; error bars, standard error of the mean. Source data are provided as a Source Data file. **c** Ratio of GEP1/GEP2 average gene signature expression in low-risk UM cells (92.1, blue) and high-risk UM cells (MP38, red) upon 48-h treatment with PRT, reversine (CIN induction) or UMK57; FPKM values obtained from bulk RNA-seq are reported (bar, mean; bars, standard deviation). Biological triplicates. **d** Schematic of proposed model: Loss of PRC1 ligase activity leads to transcriptional de-repression of target genes contributing to GEP2 phenotype; this concomitantly promotes nuclear enlargement and morphological changes toward an epithelioid phenotype, and enhances migration through CIN-induced STING signaling. Pharmacologic modulators shown in yellow; (PRT, PRC1 inhibition; UMK57; CIN suppression; H151, STING inhibition). CIN, chromosomal instability.

Chronic activation of noncanonical NF-κB and inflammatory pathways downstream of cGAS-STING in cancer cells with CIN has been shown to promote migration and metastasis^56^. We thus asked whether PRC1 loss could promote a migratory phenotype mediated by downstream CIN induction. Indeed, PRT-treatment enhanced the migration of low-risk UM cells, but not high-risk UM cells (Fig. 8a and Supplementary Fig. 10c). To determine whether the migratory phenotype induced by PRC1 inhibition is mediated through CIN and STING, we employed pharmacologic modulators of CIN and STING. CIN was suppressed by de-stabilizing kinetochore-microtubule attachments using UMK57, a small molecule that has been proposed to potentiate the activity of MCAK, a kinesin-13 protein whose microtubule-destabilizing activity at the centromere is critical for faithful chromosome segregation^58–60^. Treatment with UMK57 completely rescued the increase in chromosome missegregation seen upon PRT treatment of 92.1 low-risk UM cells and significantly reduced their migration (Supplementary Fig. 11a and Fig. 8a). We next used H151, a small molecule covalent inhibitor of STING that blocks its activation-induced palmitoylation^61^. Treatment with H151 also rescued the migratory phenotype seen upon PRC1 inhibition in low-risk 92.1 cells (Fig. 8b), indicating that CIN- and STING-mediated migratory effects are downstream of PRC1 loss. These drug treatments inhibited migration more than the DMSO control, which we attribute to low levels of basal missegregation rates even in low-risk DMSO-treated 92.1 cells.

Finally, we set out to determine the relative contributions of CIN to the transcriptional profile that defines high-risk UM. We performed RNA-sequencing on low-risk 92.1 cells with and without CIN induction through reversine treatment, a potent inhibitor of the mitotic kinase Mps1^62^ in the absence of PRC1 inhibition. Pharmacologic CIN induction led to an expected increase in chromosome missegregation and micronuclei formation (Supplementary Fig. 11a, b), as well as transcriptional changes accounting for half of genes (50.4%) that were differentially expressed upon PRC1 inhibition (Supplementary Fig. 11c), including upregulation of inflammatory and migratory pathways (Supplementary Fig. 11d). However, it had no effect on the expression of GEP prognostic genes (Fig. 8c). Accordingly, CIN suppression using UMK57 in high-risk MP38 cells, or in 92.1 cells treated with PRT, did not alter the GEP-specific gene expression signature, despite leading to a decrease in chromosome missegregation and micronuclei formation (Fig. 8c and Supplementary Fig. 11a, b). Collectively, these findings indicate that while the migratory phenotype induced by PRC1 loss is mediated through CIN, canonical GEP-related genes are likely under direct control of PRC1, independent of CIN (Fig. 8d).

## Discussion

Our results provide mechanistic insight into UM progression and argue against a model of independent clonal origins, implying that UM with good prognosis, left untreated, may evolve over time to acquire a more aggressive phenotype. Tumor stratification based on bulk transcriptional profiling of an inherently heterogeneous tumor is likely biased by detection of the most common tumor cell subpopulation or the region sampled during fine needle aspiration. Surprisingly, we observed that UMs with *BAP1* loss exhibited de-repression—rather than enhanced repression—of PRC1 target genes, and loss—rather than gain—of H2AK119Ub. While BAP1 hydrolyzes ubiquitin on H2AK119Ub, thereby counteracting PRC1 action, its role in modulating the expression of genes under Polycomb regulation is less defined. For instance, in human cells, *BAP1* appears to safeguard against gene silencing mediated by PRC1^16^, whereas in *Drosophila*, loss of *BAP1* homologue, *Calypso*, or other components of PR-DUB, was also found to lead to de-repression of Polycomb target genes^38,63^. It is possible that an intricate balance between H2A ubiquitination and de-ubiquitination may be necessary to achieve target gene repression^64^ and that BAP1 loss might be necessary in order to maintain very low basal levels of H2AK119Ub, which might be required for cellular viability. *BAP1* function may also depend on cellular context. For instance, *BAP1* loss mediates apoptosis in fibroblasts, liver, and pancreatic cells but not in melanocytes and mesothelial cells^65^. Our results show that, in addition to *BAP1* loss, high-risk tumors exhibited loss of core PRC1 components and decreased H2A ubiquitination, indicating that *BAP1* loss in UM reflects broader dysfunction in loss of PRC1-mediated transcriptional repression. Our results are in line with other studies that have demonstrated aberrant expression of epigenetic modifiers in high risk UM^66^, significant intra-tumoral spatial heterogeneity in H2AK119Ub immunostaining of UM, and reduced H2AK119Ub staining in UM compared to normal choroid^67^.

More generally, this work links two important hallmarks of tumor progression by demonstrating a functional link between epigenetic reprogramming and CIN, both of which have been implicated in tumor progression and metastasis^56,68^. In addition to its role in transcriptional regulation, Polycomb group proteins interact to form higher order chromatin structure. Consequently, their disruption changes the three-dimensional genome topology and may contribute to CIN^15,69–71^. In mouse fibroblasts, loss of canonical PRC1 component, CBX2, induces CIN^72^. Indeed, we found that inhibition of PRC1—but not PRC2—promotes widespread CIN in UM cells. We also found that PRC1 inhibition promotes a transition toward an epithelioid morphology characterized by nuclear enlargement, thereby linking epigenetic, transcriptional and histological features of UM. In line with our findings, nuclear enlargement has recently been shown to occur secondary to *RING1B* (*RNF2*) knockdown in mouse embryonic stem cells^69^.

It is crucial to identify pathways downstream of PRC1 loss that contribute to metastasis and which can be targeted for therapeutic benefit. We recently demonstrated that CIN can drive metastasis in a tumor cell-autonomous manner, through aberrant activation of the cytosolic DNA-sensing cGAS-STING pathway^56^. Here, we found that PRC1 inhibition triggers chromosome segregation errors that promote pro-metastatic chronic inflammatory signaling. While the migratory phenotype induced by PRC1 inhibition was suppressed in the presence of small molecule inhibitors of either CIN or STING, canonical GEP-related gene sets that define aggressive UM in the clinic appear to be under direct PRC1 control Suppression of CIN was insufficient to alter their expression levels. This suggests that PRC1 deregulation has both CIN- *dependent* and *independent* mechanisms of promoting UM progression. Nonetheless, given its functional consequences on cell migration, modulation of pathways downstream of PRC1, like CIN and the cGAS-STING pathway, may still represent a promising target that could be exploited to suppress UM progression and metastasis. By uncovering key steps involved in UM progression, our work highlights an opportunity for earlier therapeutic intervention to suppress tumor evolution toward a lethal metastatic phenotype.

## Methods

### Human specimens

Primary uveal melanomas were obtained from patients undergoing enucleation surgery at Memorial Sloan Kettering Cancer Center (MSKCC, New York, NY) after obtaining informed consent. All protocols adhered to the tenets of the Declaration of Helsinki and were conducted in accordance with the regulations of the Health Insurance Portability and Accountability Act. Internal Review Board (IRB) approval was obtained from Memorial Sloan Kettering Cancer Center, NY (IRB protocol #17-206). Samples were collected from 6 patients spanning prognostic gene expression profiles (GEP1 and GEP2). Patient gender, primary lesion size, diagnostic stage, and disease progression, as well as copy number alterations, UM hotspot and BAP1 mutations identified by targeted exome sequencing using MSK-IMPACT (performed on formalin-fixed, paraffin embedded enucleation specimens)^22^ are annotated in Supplementary Table 1. Immediately after enucleation, a sclera window was made and tumor was removed (with a freer elevator) and sent for DecisionDx for bulk GEP classification and for scRNAseq processing. Tissue samples were immediately placed in RPMI media (Corning) or Hypothermosol on ice and dissociated using both mechanical and enzymatic digestion (Human Tumor Dissociation Kit #130-095-929, Miltenyi Biotec), generally within 1 h of surgical resection. Tissues were minced with a razor blade in the Miltenyi enzyme mix according to the manufacturer’s specifications and transferred to a Gentle MACS Octo Dissociator with heaters (# 30-096-427, 37 °C) for further mechanical dissociation. Upon completion, cell suspensions were passed through a 70 µm filter and washed twice with FACS buffer (2% heat-inactivated FBS, 1 mM EDTA and Pen/Strep in PBS without Ca or Mg). Red blood cells were lysed in Red Blood Cell Lysis Solution (ACK lysis buffer) once or twice depending on red blood cell content, and final single-cell suspensions were made in Hanks’ Balanced Salt Solution (HBSS). For scRNA-seq, the remaining cell suspensions were subsequently flow sorted with a BD FACSAria II cell sorter fitted with a 100 µM nozzle to enrich for viable, single cells according to forward and side scattering, and DAPI exclusion. Cells were sorted directly into RPMI media with 10% FBS, washed thrice and re-suspend in PBS with 0.04% BSA for single cell encapsulation. Final cell concentrations were determined with a hemocytometer.

### scRNA-seq library generation (inDrops protocol)

Barcoded hydrogel bead synthesis, single cell encapsulation and library construction were done following a modified inDrops protocol^73,74^. Barcoded hydrogel beads were synthesized in house and contained a poly(T) sequence, T7 promoter, PE1 sequencing primer, unique cell barcode, unique molecular identifier (UMI) and a photo-cleavable linker. Cells were re-suspended in PBS with 0.04% BSA at ~400 cells/µL; average viability of loaded cells was 87%. Before encapsulation, cells were mixed at a 1:1 ratio with OptiPrep mix (32% OptiPrep Density Gradient Medium (Sigma-Aldrich), 0.08% BSA in PBS). Single cells were encapsulated in droplets together with barcoded hydrogel beads and reverse transcription mix (SuperScript™ III Reverse Transcriptase, SUPERase-In, FS buffer (Thermo Fisher Scientific) NP-40, DTT, MgCl_2_, Tris and dNTPs) using a microfluidic device with three inlet channels for aqueous solutions (barcoded hydrogel beads, RT mix and cells), an oil inlet, and an outlet channel for emulsion. Emulsion was collected for 30 min in a 1.5 mL tube in ice block. Immediately after encapsulation, primers were released from hydrogel beads by incubating the emulsion on ice under UV light for 7 min. The emulsion was then transferred to heat block at 60 °C for 1 min, 50 °C for 2 h (RT), followed by heat inactivation at 70 °C for 15 min. The emulsion was then divided into smaller aliquots that contained an estimated 5000 cells. A few drops of 20% 1H,1H,2H,2H-perfluorooctanol (PFO, Sigma-Aldritch) was added on top of each tube to break emulsions, which were then transferred and stored at −80 °C. Hydrogel beads were removed by filtration through a spin column (Zymo Research) and excess primers were digested using Exo1, HinF1, and FastAP enzyme mix (Themo Fisher Scientific). After the DNA/RNA duplex was purified using 1.2X SPRIselect beads (Beckman Coulter), second strand synthesis (SSS) was done using NEBNext® Ultra™ II Non-Directional RNA Second Strand Synthesis Module (New England BioLabs) at 16 °C for 2.5 h followed by inactivation at 65 °C for 20 min. SSS reaction material was then amplified using HiScribe™ T7 High Yield RNA Synthesis Kit (New England BioLabs) for 15 h at 37 °C. Reaction products were purified using 1.2X SPRIselect beads (Beckman Coulter) and their quality was evaluated on Agilent Bioanalyzer 2100. Amplified material was fragmented using RNA fragmentation reagents (Ambion Life Technologies) at 70 °C for 2.5 min. Fragmentation was stopped by addition of ice cold 1.2X SPRIselect beads mixed with STOP solution. After purification, fragmented aRNA was mixed with PE2-STUB (IDT) which contains random hexamers, incubated for 3 min at 70 °C, cooled on ice, and reverse transcribed using PrimeScript RTase (Takara Bio USA) for 60 min at 42 °C. Libraries were purified with 1.2X SPRIselect beads (Beckman Coulter) and amplified via PCR (Kapa 2× HiFi HotStart PCR mix, Kapa Biosystems) using P1-P2 Illumina index primers; optimal cycle number was determined using qPCR. Amplified and indexed libraries were cleaned two times using SPRIselect double-sided size selection (0.6X and 0.8X). Library size was analyzed using Agilent Bioanalyzer 2100 and quantified by Qubit dsDNA HS Assay kit (Thermo Fisher Scientific). Libraries were sequenced one per lane of HiSeq2500 (Illumina) paired-end read flow cell, loaded at a 10.5pM concentration with 15% PhiX spike-in. 54 bp were sequenced in the forward read (inDrop Barcode + UMI) and 46 bp on the reverse read.

#### scRNA-seq computational analysis

*Pre-processing, cell selection and filtering*. The Sequence Quality Control (SEQC) package^32^ was utilized to process the data, constructing a count matrix from raw reads, including de-multiplexing, alignment, error-correction, and the generation of a raw digital expression matrix by collapsing groups of reads with the same unique molecular identifier (UMI), cell barcode and gene annotation as previously described^75^. Alignment to the hg38 annotation was restricted to transcribed, polyadenylated RNA of length >200 nucleotides (gene biotypes accessible by 3ʹ mRNA sequencing technologies) to increase mapping specificity. SEQC then follows with a number of filtering steps to ensure data quality. Viable cells were distinguished from droplets consisting of ambient mRNA transcripts arising in solution due to premature lysis or cell death based on library size; whereby cells were filtered beyond the knee point of the second derivative of the empirical cumulative density function of total cell transcript counts (Supplementary Fig. 1a). In addition, cells with low complexity libraries (in which detected transcripts are aligned to a small subset of genes) were filtered (Supplementary Fig. 1b). Cells with >25% of transcripts derived from mitochondria were considered apoptotic and also excluded (Supplementary Fig. 1c). This yielded a total of 17,074 patient-derived cells with a median library size of 1619 transcripts per cell, for downstream analysis. The number of transcripts and unique genes detected per library were highly reproducible across patients (Supplementary Fig. 1d, e). Initially, library batch effects were ruled out by analyzing two independent single cell libraries prepared from the same patient, UM01 (Bio Rep A and B). Finally, genes detected in fewer than 10 cells or genes with low expression levels, identified as those with count values <5 standard deviations from the second mode of the log-log distribution of total transcript counts/gene, were excluded, yielding a filtered count matrix with 17,074 cells and 14,642 genes for downstream analysis.

*Normalization and imputation*. The filtered count matrix was normalized for library size per cell, whereby the expression level of each gene was divided by the cell’s total molecule counts and then scaled by the median molecule counts of all cells (i.e., normalized count matrix). Principle Component Analysis (PCA) was then computed using randomized principal component analysis^76^ applied to the normalized count matrix. Subsequently, ordinary least squares was applied to linearly regress library size out of each principle component because a partial correlation was observed between some principle components and cell size. Finally, MAGIC imputation^34^ was applied to the median-normalized count matrix to further denoise and recover missing gene values using conservative parameters (*t* = 3, *k* = 27). Imputation was performed using the first 20 principle components of the normalized count matrix (accounted for >60% of variance in the data) and yielded the imputed count matrix.

*Data visualization*. The global atlas of all patient cells, including tumor, immune and photoreceptor cell subpopulations (Supplementary Fig. 1) were visualized using the Barnes-hut approximate version of t-SNE^77^ (https://github.com/lvdmaaten/bhtsne) computed on the principle components of the imputed count matrix. This visualization was appropriate given the diversity of cell types represented in these data subsets. Force-directed graphs^78^ were alternatively used to visualize tumor cell states, which better represent cell state transitions while maintaining a coherent global structure (Fig. 1d, Supplementary Fig. 4a). Force-directed layouts were computed on the principle components of the imputed count matrix using a k-NN graph^23^; whereby the adjacency matrix representing this k-NN graph was converted to an affinity matrix using an anisotropic Gaussian kernel^34^ to better account for large differences in data density. This adaptive kernel used distance to the *k* = 12 neighbor as the scaling factor for each cell to determine the affinities^34^. The affinity matrix was then used as input to compute the force-directed layout using the ForceAtlas2 python module^78^. For both visualization methods, the number of principle components was selected per dataset (global atlas of all patient cells and tumor cells subset) based on the knee point of cumulative explained variance. Principle component analysis re-applied to the post-imputation count matrix yielded 33 and 41 principle components explaining ~90% of variance in the global atlas of all patient cells and in the tumor cell subset alone respectively.

*Selection of variably expressed genes*. Variably expressed genes were selected from single cell data based on the standard deviation of their expression across all cells. The distribution of the standard deviation per gene across all cells, for all genes, was fit to a bi-modal distribution using least squares minimization. Highly variable genes were identified as those whose standard deviation was greater than the second mean of this distribution less 1.5X its standard deviation.

*Phenograph clustering and cell type annotation*. Phenograph clustering^23^ was computed directly on the top 33 principle components of the imputed computed count matrix for all merged patient cells. The number of nearest neighbors, *k*, used to construct the k-NN graph was selected such that the Jaccard graph per data subset was fully connected (*k* = 8) and it was within a range of *k* for which cluster assignments were robust. Robustness of Phenograph clusters was evaluated by computing the adjusted rand index (ARI) between categorical cluster assignments made using all pairwise values of *k*. For each pairwise comparison, we compute the ARI across all cells. The resulting *k*-by-*k* matrix of ARI values was visualized as a two-dimensional heatmap (Supplementary Fig. 1f). Clustering was performed with *k* = 35 because this was the minimum value of *k* for which categorical assignments were stable (ARI > 0.75). This yielded 27 Phenograph clusters (Supplementary Fig. 1g) in the merged atlas of all single cells acquired from six patients (Supplementary Fig. 1h). Phenograph clusters were then hierarchically clustered by their imputed expression of canonical markers, z-normalized across cells, to inform gross cell type assignments (i.e., tumor, immune subsets, and photoreceptors). Tumor cells expressed genes consistent with a melanocytic cell of origin; whereas photoreceptors and immune subpopulations were distinguished by expression of *RHO* and *CD45* respectively (Supplementary Fig. 1i).

Cell type assignments were further validated by examining the genome-wide correlation between Phenograph cluster expression medians and the expression profiles of sorted immune populations, for which bulk microarray and RNA-seq datasets were available^79,80^. First, bulk microarray datasets were log_2_ transformed and library-size normalized. Then, the mean expression per cell type was computed across biological replicates and centered by mean subtraction per gene across all cell types. Correspondingly, the median imputed expression of each gene per Phenograph cluster was computed and likewise centered by subtracting the mean expression per gene across all clusters. Finally, the correlation between the centered transcriptional profile of each Phenograph cluster and bulk immune cell type was computed in a pairwise manner using all genes variably expressed in single cell data and detected in bulk immune data (*n* = 5480 genes). For each pairwise correlation, the Python package scipy was utilized to compute a *p* value testing for non-correlation. Hierarchical clustering of the Pearson correlation between each Phenograph cluster and bulk immune cell type is shown in Supplementary Fig. 1j. Only correlation coefficients with absolute magnitude >20% and characterized by *p* < 0.01 are visualized; all others are whited out. Phenograph clusters (columns) not meeting these criteria are not displayed. Expectedly, tumor cell clusters do not show a significant correlation with immune subsets.

*Copy number variation analysis*. The inferCNV package^81^ was used to predict large-scale copy number variants (CNV) based on average gene expression across chromosomes for each patient. Control normalization was performed relative to a diploid reference (all *CD45* + immune cells) from the tumor expression values. Standard use of the algorithm was applied with the following parameter selections: (1) mean gene cutoff across all cells was set to 0.05, (2) the three state Hidden Markov Model (HMM) was used for predicting large-scale CNV events and (3) the subcluster analysis method was used to account for intra-tumor heterogeneity, whereby subclusters were partitioned using the random trees method. Low-probability CNV predictions were filtered out using the Bayesian network latent mixture model with a normal probability threshold of 0.6 for assigning putative CNV events. Modified gene expression along chromosomes for all tumor cells clustered by the Ward distance metric are visualized as heatmaps (Supplementary Fig. 2a). The fraction of tumor cells in which canonical UM alterations were detected by the HMM are summarized per patient in Supplementary Fig. 2b. Phylogeny of intra-patient subclones detected by inferCNV were reconstructed for tumor subclones detected in >5% of the patient tumor based on the hamming distance metric (Supplementary Fig. 2c). The length of each branch represents the Hamming distance between each child and parent leaf, where the root of the tree is a diploid cell. Branch width and node size are scaled by the fraction of cells in each subclone (plus a minimal constant), where intermediate branch widths are dependent on the sum of downstream subclones. Tumor cells showed distinct subclonal patterns of copy number aberrations, including characteristic loss of chromosome 3 and gain in chromosome 8.

*Clinical prognostication of individual tumor cells*. Tumor cells were directly subset from the global atlas of all patient cells based on the cell type annotations described above (*n* = 16,077 tumor cells with median library size of 1680 transcripts). Normalization, imputation and visualization were computed for the tumor subset separately, as described above, from the level of raw counts. Individual tumor cells were then assigned to low-risk UM (Gene expression profile 1, GEP1) and high-risk UM (Gene expression profile 2, GEP2) according to their mean imputed expression of characteristic genes used clinically to stratify patients (Supplementary Data File 1). A two-component Bayesian Gaussian Mixture Model (BGMM) was fit to this two-dimensional distribution (Supplementary Fig. 3a, b), computed using the diagonal covariance matrix of each mixture component using the python package *sklearn*. The BGMM model was then used to predict the most probable clinical prognostication for input cells, where the k-means search was initialized with a fixed seed for reproducibility. To control for difference in patient cell numbers, estimations of GEP fractions per patient were generated in subsets of tumor cells (*n* = 500) randomly sampled over 20 rounds (Fig. 1c). In a similar manner, individual tumor cells were alternatively assigned to one of the four TCGA molecular subtypes according to mean imputed expression of their characteristic genes (Supplementary Data File 1) using a four-component BGMM. The only exception being that the four-component model used spherical covariance instead of diagonal covariance, so that each component has its own single variance. The most probable TCGA molecular subtypes were assigned to each tumor cell and visualized on a t-SNE plot, generated using the same mean imputed expression of characteristic genes (Supplementary Fig. 3e) and on a two-dimensional scatter plot reflect the average expression of the GEP1 and GEP2 gene signatures per cell (Supplementary Fig. 3f). Expectedly, TCGA subtypes 1 and 2 correlate with high GEP1 expression and conversely, TCGA subtypes 3 and 4 correlate with high GEP2 expression.

*Defining phenotypic volume*. To quantify the extent of transcriptional complexity within each patient tumor (Fig. 1f), we used a metric of Phenotypic Volume defined in Azizi et al.^32^ as the pseudo-determinant of the gene-gene covariance matrix within each subgroup. This metric for Phenotypic Volume considers covariance between all gene pairs, in addition to their variance, to quantify the volume spanned by independent cell states. Practically, it is the pseudo determinant of the covariance matrix, which can be computed as the product of its nonzero eigenvalues; here, the gene-gene covariance matrix was computed across all variably expressed genes (*n* = 12,755). Number of cell states, and therefore Phenotypic Volume, naturally correlates with population size. Therefore, to correct for the effect of cell number differences across GEP1 and GEP2 groups when comparing their Phenotypic Volume, the same number of cells (*n* = 150) was randomly subsampled from each prognostic group in the comparison. The gene-gene covariance matrix was then empirically computed per random subset of cells on the normalized, but un-imputed data. Imputation was not utilized here because it alters the gene-gene covariate structure. The log Phenotypic volume was then computed as the sum of the log of the non-zero eigenvalues, ${\lambda }_{e}$, of each empirical gene-gene covariance matrix:

$${{\log }}\left({{{{\mathrm{{Phenotypic}}}}}}\,{{{{\mathrm{{Volume}}}}}}\right)=\mathop{\sum}\limits_{e}{0.5\ast {{\log }}}_{10}\left({\lambda }_{e}^{2}\right){{{{{\rm{;}}}}}}\,\forall \,{\lambda }_{e} > 0.$$

Finally, given the high number of dimensions (genes), the log of the Phenotypic Volume was normalized by the total number of genes. Subsampling was repeated 100 times to achieve the range of Phenotypic Volume across patients reported in Fig. 1f.

*Multi-scale diffusion distance*. Diffusion maps were used to characterize major components of variation across cells^82^. As described in Van Dijk et al.^34^, a cell-cell Euclidean distance matrix was computed based on the principle components of the normalized (unimputed) count matrix, where the number of principle components was selected based on the knee point of the cumulative explained variance. An adaptive Gaussian kernel was then applied to convert distances into affinities, so that similarities between two cells decreases exponentially with distance. The affinity matrix was then row-normalized to construct a Markov transition matrix, whose eigenvectors are termed diffusion components. The eigenvalues of this matrix provide information on the importance of each diffusion component. The pairwise distance between cell *i* and cell *j* can be computed as the sum of the Euclidean distances between diffusion components (e_*i*_, e_*j*_), scaled by their eigenvalues (${{{{{\rm{\lambda }}}}}}$) and powered by the number of diffusion steps, *t*.

$${{{{{{\rm{D}}}}}}\left({{{{{{\rm{e}}}}}}}_{{{{{{\rm{i}}}}}}},{{{{{{\rm{e}}}}}}}_{{{{{{\rm{j}}}}}}}\right)}^{2}={\sum }_{{{{{{\rm{l}}}}}}=1}^{{{{{{\rm{L}}}}}}}{{{{{{\rm{\lambda }}}}}}}_{{{{{{\rm{l}}}}}}}^{2{{{{{\rm{t}}}}}}}{({{{{{{\rm{e}}}}}}}_{{{{{{\rm{l}}}}}},{{{{{\rm{i}}}}}}}-{{{{{{\rm{e}}}}}}}_{{{{{{\rm{l}}}}}},{{{{{\rm{j}}}}}}})}^{2}$$

where *t* represents the number of steps through the graph and ${{{{{{\rm{e}}}}}}}_{l,i}$ represents the embedding of cell *i* along diffusion component *l*. To avoid setting a particular *t* and to render the distance robust to outlier cells and density differences, we used the multi-scale approximation to this distance described in Setty et al.^83^. The dimension *L* of the embedding was chosen to be 5 based on the Eigen gap among the top Eigenvectors.

*Tumor cell archetypal analysis*. To define tumor cell archetypes we applied the Principle Convex Hull Analysis (PCHA) method^84^ to the top principle components of the imputed count matrix; where 22 principle components were selected based on the knee point of their cumulative explained variance. Dimensionality reduction was applied to improve robustness of the archetypal analysis, since volume and therefore number of data points needed to approximate the bounding convex-hull grows exponentially with dimensions. Using the first 22 principle components for the PCHA method, we searched for 8 archetypes whose convex-hull closely approximates the data. The optimal number of archetypes used to define the bounding convex-hull was identified by identifying the knee point of variance explained by the model as a function of archetype number. The goal of archetypal analysis is to identify an optimal set of archetypes so that their convex combination best re-approximates the data points. Because the archetypes must lie near the convex hull of the data, they represent its extreme phenotypic states. To characterize these extreme phenotypic states, we identified the cell nearest to each archetype based on the Euclidean distance metric and then defined a soft neighborhood around each archetype based on the multi-scale diffusion distance (computed on the principle components of the un-imputed normalized count matrix, as described above); whereby cells within a specific radius of each archetype are assigned to each bounding phenotypic state. This radius was defined per archetype as ½ the multi-scale diffusion distance to its nearest archetype. This radius ensures the neighborhood surrounding each archetype spans a similar range on the manifold for each archetype. Finally, all individual tumor cells were likewise assigned to their nearest archetype according to this multi-scale diffusion distance (Supplementary Fig. 4a) to evaluate the fraction of cells closest to each archetype per patient (Supplementary Fig. 4b).

*Archetype-based differential gene expression analysis*. The soft neighborhood of cells surrounding each archetype now allows us to characterize the gene expression profiles of these bounding phenotypic states. As previously described^34^, we identify genes whose expression maximally distinguishes each archetype against background gene expression according to the earth mover’s distance (EMD)^85^; which is a non-parametric measure of distance between two distributions that quantifies the flow required to morph one distribution into another. For each archetype, a background consisting of the same number of cells is randomly sampled from all cells that are not a member of its archetypal neighborhood. For each archetype, we compute the EMD to background for each variably expressed gene. To ensure robustness we perform background subsampling and the EMD computation 100 times and report the average score per gene; allowing us to identify genes whose expression maximally distinguishes each archetype from background (Supplementary Fig. 4c and Supplementary Data File 2).

*Gene signatures for archetype annotation*. A custom annotation file was generated to probe molecular signatures defining tumor archetypes. This was assembled by integrating all gene ontology (GO) signatures related to UM (GEP1/2, monosomy 3, UM with aneuploidy), several PRC1 and 2 target genes, chromatin binding and modification, histone modification (ubiquitination, deubiquitination, and trimethylation), chromosome organization, metastasis, neural crest differentiation, retina and optic nerve development and homeostasis and protein ubiquitination, as well as complete KEGG and Hallmark Gene sets (see Supplementary Data File 3).

*Archetype-based gene set enrichment analysis*. To visualize GSEA results in an intuitive manner for archetype characterization, gene signatures distinguished by a Bonferroni corrected ${{{{{{\rm{p}}}}}}}_{{{{{{\rm{adj}}}}}}}$ < 0.25, a nominal ${{p}}$ < 0.05 and an absolute Normalized Enrichment Score ${{{{{\rm{abs}}}}}}({{{{{\rm{NES}}}}}})$ > 1.5 were considered significant and hierarchically clustered (clusters and gene signatures) by NES according to the correlation distance metric (Supplementary Fig. 4d). Areas not meeting these criteria were whited out on the heatmap. An unfiltered list of all gene signatures distinguished by ${{{{{{\rm{p}}}}}}}_{{{{{{\rm{adj}}}}}}}$ < 0.25 is provided in Supplementary Data File 4.

*Analysis of additional validation datasets*. The filtered and annotated count matrix from the independent patient cohort^24^ was received directly from its authors. This scRNA-seq dataset includes 11 patient samples – 3 metastatic and 8 primary tumor samples. Analysis was limited to cells annotated by the authors as tumor cells and genes not expressed in any tumor cells were removed, yielding a filtered count matrix with 25,141 tumor cells and 26,537 genes. Of all tumor cells, 23,100 were derived from primary tumors. Primary tumor cells were processed independently and were the focus of downstream analyses. The metastatic samples were only included when training the BGMM (as described above under clinical prognostication of individual tumor cells) because the most extreme GEP1 case in this cohort, was surprisingly a latent metastasis. As described above, filtered count matrices were normalized for library size per cell, whereby the expression level of each gene was divided by the cell’s total molecule counts and then scaled by the median molecule counts of all cells (yielding normalized count matrices). Principle Component Analysis (PCA) was then computed using randomized principal component analysis^76^ applied to the normalized count matrix. Subsequently, ordinary least squares was applied to linearly regress library size out of each principle component because a partial correlation was observed between some principle components and cell size. Finally, MAGIC imputation^34^ was applied to the median-normalized count matrix to further denoise and recover missing gene values using conservative parameters (*t* = 3, *k* = 27). Imputation was performed using the first 32 principle components of the normalized count matrix. These principle components accounted for >80% of the variance and yielded the imputed count matrix. De-repression of PRC1 target genes and upregulation of *cGAS, STING*, and downstream inflammatory response pathway effects were validated in primary tumor cells ranked by the average GEP2 gene expression signature (Supplementary Figs. 7a, 9e) using this imputed count matrix. Tumor cells were likewise assigned to a GEP or TCGA prognostic class as described above; with the exception that a tied-covariance matrix was used when fitting the four-component BGMM.

#### Cell culture

HEK293T and human UM cell lines MP41, MP38, MP46 and Mel285^35^ were purchased from the American Type Culture Collection (ATCC), and 92.1 and Mel202^36,37^ from the European Collection of Cell Cultures (ECACC). Melanoma cells were grown in RPMI-1640 media (Gibco) supplemented with 10% fetal bovine serum (FBS, Sigma), and HEK293T cells were grown in Dulbecco’s modified Eagle’s medium (DMEM, Gibco) supplemented with 10% FBS. Cell culture media were supplemented with 1% penicillin/streptomycin and cells were maintained at 37 °C and 5% CO_2_.

#### Pharmacologic treatment

Cells were grown under regular conditions until they reached ~80% confluence. Culture media (RPMI) containing the following compounds dissolved in DMSO (Fisher BioReagents), or an equal volume of DMSO, were added to the cells; 50 μM PRT (PRT4165, Sigma-Aldrich), 1 μM EPZ (EPZ011989, Cayman Chemical), 5 μM GSK126 (Xcess Biosciences), 1 μM UMK57 (Aobious), 0.5 μM reversine (Cayman Chemical) and H-151 (Invivogen). For experiments with treatment extending beyond 2 h, culture media were supplemented with 10% FBS and replenished after the first 24 h, with the applicable compound. For the long-term PRT treatment, cells were passaged under regular conditions and fresh media (RPMI + 10% FBS) with 50 μM PRT or 0.1% DMSO, were added for 48 h prior to passaging the cells, and cells were passaged 4–6 times. Images of cells were obtained using bright field microscopy (Evos, Life Technologies).

#### Lentiviral transfection

HEK293T cells were transfected with the pLentiCas9-T2A-BFP vector^86^ (Addgene plasmid # 78547; http://n2t.net/addgene:78547; RRID:Addgene_78547, deposited by Roderic Guigo & Rory Johnson), or gRNA cloned into pLenti-U6-sgRNA-PGK-Neo vector (Applied Biological Materials Inc.). gRNA were designed to target either RING1 or RNF2 and were purchased from Applied Biological Materials Inc. (Cat. 396021110195 and 402851110195). The transfection mixture consisted of the TurboFectin transfection reagent (Origene) and lyophilized packaging plasmids (Origene) dissolved in Opti-MEM. Approximately 18 h after transfection, the culture medium was changed, and the supernatant was collected 24 and 48 h post-transfection and filtered with a 0.45-μm PVDF filter (Millipore). The virus was aliquoted and stored at −80 °C.

Mel285 cells were first transfected with lentiviruses encoding the Cas9 plasmids in a 6 well plate with polybrene (Sigma-Aldrich) added to a final concentration of 8 μg/mL. At 72 h post transduction, blasticidin (InvivoGen) was added to each well to a final concentration of 6 μg/mL. Culture media was exchanged every 48 h and blasticidin selection was maintained for a total of 7 days. Next, single cell clones, Mel285.Cas9, were derived in 96 well plates using blasticidin (6 μg/mL) as the selection agent.

Mel285.Cas9 cells, derived from a single clone, were then transfected with lentiviruses encoding different gRNA, in 6 well plates with polybrene (Sigma-Aldrich) added to a final concentration of 8 μg/mL. At 72 h post transduction, G418 sulfate (Geneticin, ThermoFisher) was added at a final concentration of 1000 μg/mL. Culture media was exchanged every 48 h and G418 selection was maintained for a total of 7 days. Successful knockout of either RING1 or RNF2 was confirmed using western blot analysis (Fig. 5e).

#### Bulk RNA-seq

Total RNA was the extracted using the QIAShredder and the RNeasy extraction kit (Qiagen) and sequenced on NovaSeq (Illumina) to generate 150 bp pair-end reads. Raw reads (FASTQ) were aligned to the human reference genome (GRCh38) using STAR on Partek Flow software, version 8.0. Aligned reads were mapped to transcripts (Ensembl Transcripts release 102) using Partek’s modified expectation-maximization (EM) algorithm for transcript quantification^87^, with strict paired-end compatibility. Gene counts were normalized per sample to generate fragments Per Kilobase of transcript per Million mapped reads (FPKM) values. Differential gene expression analysis was performed using the gene specific analysis (GSA) tool on Partek, which is based on the Limma + voom package^88^. Gene levels distinguished by FDR value <0.05 were considered significant. Genes not meeting these criteria were displayed in gray in the volcano plots in Fig. 3a. A full list of the genesets used in Fig. 2e–g and Fig. 3e–g and Supplementary Fig. 7d–f and Fig. 8f is annotated in Supplementary Data File 1. PCA was computed using Partek Flow software using the normalized gene counts for GEP1 and GEP2 genes or TCGA subtypes^24^ (gene lists in Supplementary Data File 1) and applied to UM cells. To visualize how different UM cells fall on the GEP or TCGA-subtypes spectrum, cells were plotted as a function of the first and second principal components of the GEP 1 and GEP2 genes or the genes defining the 4 TCGA subtypes (Supplementary Fig. 5a, b). GSEA was performed using the GSEA software (The Broad Institute) using the gene sets in Supplementary Data File 3. To visualize GSEA results in an intuitive manner, relevant gene signatures were plotted as a function of their NES and -Log_10_(FDR) values (Fig. 5a, Supplementary 9a and 11d). Complete GSEA results for UM cells and upon PRT or reversine treatment, including nominal p-values, are listed in Supplementary Data Files 5–7.

#### Gene expression analysis using NanoString

Biological triplicates from low-risk UM cells (92.1) upon 24 h DMSO or EPZ-treatment, or high-risk UM cells (MP38) upon 24 h DMSO-treatment were collected, and RNA was extracted using the QIAShredder and the RNeasy extraction kit (Qiagen). 100 ng of RNA per sample was prepared for analysis with a NanoString Human Custom Panel (gene lists in Supplementary Data File 8) chip. The assay was performed on an nCounter MAX Analysis System (Stem Cell Genomics and Microscopy Core, Sanford Consortium for Regenerative Medicine, La Jolla) according to the manufacturer’s instructions. Data was then normalized and analyzed by ROSALIND® (https://rosalind.onramp.bio/), with a HyperScale architecture developed by OnRamp BioInformatics, Inc. (San Diego, CA) to interpret targeted gene expression data. Read Distribution percentages, violin plots, identity heatmaps, and sample MDS plots were generated as part of the QC step. The limma R library^89^ was used to calculate fold changes and p-values and perform optional covariate correction. Results are shown in Supplementary Fig. 8e.

#### Chromatin profiling using CUT*&*RUN

In situ chromatin profiling of 92.1 and MP38 cells was performed using Cleavage Under Targets and Release Using Nuclease, CUT&RUN, according to Skene et al.^90^ using the CUTANA kit (EpiCypher, 14-1048). Briefly, adhered cells were scrapped off the culture plates and centrifuged at 600 × *g* for 3 min. Pellets were re-suspended twice in wash buffer, and then mixed with Concanavalin A (ConA) conjugated paramagnetic beads (EpiCypher, 21-1401). Following a 10 min incubation on a magnet rack at RT, the supernatant was discarded. Antibodies diluted in antibody buffer were added to the beads along with the bound cells as follows, H2AK119Ub (Cell Signaling, 8240), 1:100; RING1 (Cell Signaling, 13069), 1:100; RNF2 (Cell Signaling, 5694), 1:100; BMI1 (Cell Signaling, 5856), 1:100; H3K27me3 (Thermo Fisher, MA5-11198), 1:100; IgG (Thermo Fisher, MA5-11198), 1:100. Following an overnight incubation at 4 °C, the supernatant was removed, and beads were washed in digitonin buffer for a total of two washes. CUTANA pAG-MNase (EpiCypher, 15-1016) was added to the samples for 10 min at room temperature, followed by two washes in digitonin buffer. CaCl_2_ was added to a final concentration of 2 mM in order to activate MNase and initiate chromatin cleavage. Targeted digestion was performed for 2 h on ice until STOP buffer was added. Cells were then incubated at 37 °C for 10 min to release cleaved chromatin fragments. DNA was purified using the CUTANA DNA Purification Kit (EpiCypher) and eluted in 12 µL Elution Buffer. The purified DNA was quantified using Qubit 4 fluorometer (Invitrogen) as per manufacturer’s instructions. Library preparation was performed using the NEBNext Ultra II Library Prep Kit for Illumina (New England BioLabs, E7103S) per manufacturer’s instructions with minor modifications. The NEBNext Adaptors were diluted 1:10 in NEBNext adaptor dilution buffer (New England BioLabs, B1430S). Following adapter ligation, DNA cleanup was performed using 1.7x AMPure XP beads (Beckman Coulter Inc. #A63881). PCR was performed using unique dual index primer pairs (NEBNext Multiplex Oligos for Illumina, New England BioLabs, E6440S) according the following parameters: 45 s at 98 °C to activate hot-start Q5 polymerase, followed by 15 s at 98 °C, 10 s at 65 °C for a total of 13 cycles, and finally 5 min at 65 °C for final extension. DNA cleanup was performed using 1.2x AMPure beads (Beckman Coulter Inc. #A63881) and the DNA was eluted in TE buffer. DNA quantification was performed using Qubit (Invitrogen), and fragment sizes of individual libraries were analyzed on the 4200 TapeStation system (Agilent) using a DNA 1000 High Sensitivity Screentape. Libraries were pooled to a final concentration of 23 nM and sequenced on NovaSeq 6000 (Illumina), 100 bp paired-end reads.

Raw reads (FASTQ) were aligned to the human reference genome (Ch38), using STAR on Partek Flow software, version 10.0 (Partek). Genomic peaks were called using MACS2 with the following parameters (BAM, cutoff q-value 0.05, no down-sampling). A list containing H2AK119Ub and H3K27me3 peaks normalized to IgG in 92.1 cells can be found in Supplementary Data File 1. For heatmap and intensity plot representation of CUT&RUN signal, TSS plots were generated using Partek Flow software, version 10.0 (Partek) considering the region ± 5 kb around the center of the peak.

#### Immunofluorescence microscopy

Cells cultured on coverslips (Fisher Scientific) were fixed with ice-chilled (−20 °C) methanol for 15 min at −20C and subsequently permeabilized with TBS-BSA- 0.5% Triton for 5 min, and then washed with TBS-BSA. Primary antibodies for centromere (Antibodies Incorporated, 15-234-0001), cGAS (Millipore, HPA031700) or Ubiquityl-H2AK119 (Cell Signaling, 8240) diluted in TBS-BSA 1:1600 were added to the coverslips. DAPI was added along with secondary antibodies. Cells were mounted with Fluoro-Gel (Electron Microscopy Sciences) and visualized using Nikon Eclipse Ti2 microscope (60x magnification) except for cGAS and H2AK119Ub visualization which was done using Zeiss LSM 880 microscope (100x magnification for cGAS and 60x magnification for H2AK119Ub). Number of micronuclei per 60X HPF were counted in at least 10 different sections per slide. For chromosome missegregation scoring, at least 50 cells undergoing anaphase were identified and scored for missegregation. Lagging chromosomes were defined as one or more area of DAPI staining isolated between the segregating chromosomes. Acentric fragments were defined as similar to that of lagging chromosomes, but without centromere-positive staining on the lagging chromosome. Multipolar anaphases were defined as more than two sites of chromosome segregation. Chromatin bridges were defined as continuous band of DAPI staining between the segregating chromosomes. Mixed patterns were defined as a mixture of two or more of the above categories. Images of DAPI or H2AK119Ub were binarized and intensity of H2AK119Ub normalized to DAPI was measured using ImageJ (NIH) (Supplementary Data File 9).

The immunofluorescence detection of H2AK119Ub and STING in patient tissue samples were performed at Molecular Cytology Core Facility of Memorial Sloan Kettering Cancer Center using Discovery XT processor (Ventana Medical Systems). The tissue sections were blocked for 30 min in 10% normal goat serum 0.2% BSA in PBS. The primary antibody incubation was done with these antibodies: H2AK119Ub: Cell Signaling, 8240 (1:1600 dilution); and STING: Cell Signaling, 13647 S (1:100 dilution). The incubation with the primary antibody was done for 5 h, followed by 60 min incubation with either anti biotinylated anti rabbit IgG (Vector Labs, catalog#: PK6101) in 1:200 dilution. The detection was performed with Blocker D, Streptavidin-HRP D (Ventana Medical Systems), followed by incubation with Alexa Fluor™ 488 Tyramide SuperBoost™ Kit, streptavidin (Life Technologies, catalog#: B40932).

#### Nuclear size

Cell cultures were trypsinized, resuspended in pre-warmed 0.075 M KCl, incubated for 15 min at 37 °C and fixed in methanol-acetic acid (3:1). The fixed cell suspension was then dropped onto slides, stained and mounted in ProLong Gold antifade reagents with DAPI (Invitrogen). Nuclei stained with DAPI were captured using the Nikon Eclipse E800 epifluorescence microscope. Images of nuclei were binarized and nuclear size was measured using ImageJ (NIH).

#### Cell migration assay

Cells were split into 10 cm^2^ plates with RPMI + 10% FBS. Two days before cells reached 90-100% confluency, cell media were changed to media containing 50 μM of PRT, 0.5 μM reversine, 1 μM UMK57, 1 μM H-151, a combination of 50 μM of PRT and 1 μM UMK57, a combination of 50 μM of PRT and 1 μM H-151, or an equal volume of DMSO. For the combined treatments (PRT and UMK57 or PRT and H-151) UMK57, or H-151, were added 2 h prior to the PRT treatment, and supplemented with 50 μM PRT during the treatment. The following day the media was exchanged with fresh media supplemented with the same corresponding compounds. A day later, cells at 90–100% confluency were treated with mitomycin C (10 μg ml^−1^) for 1 h and then placed in media with 1% FBS. A p200 pipette tip was used to create a linear wound. Images were taken immediately (day 0), and at 24 h interval. The wound healing tool macro in ImageJ (NIH) was used for quantification of wound surface area in pixels.

#### Western blotting

Cell were lysed using RIPA buffer supplemented with Halt Protease and Phosphatase Inhibitor Cocktail (ThermoFisher Scientific). Total protein concentration was determined using BCA protein assay kit (ThermoFisher Scientific) and 10–20 μg of total protein were loaded per lane. Proteins were separated by electrophoresis on 4%–12% NuPAGE Bis-Tris Mini Gel (Invitrogen), and transferred to nitrocellulose membranes using the Trans-Blot Turbo Transfer System (Bio-Rad). Membranes were incubated in primary antibodies at 4 °C overnight diluted as follows, BAP1 (Santa Cruz Biotechnology, sc-28383), 1:100; H2AK119Ub (Cell Signaling, 8240), 1:2000; RING1/RING1A (Cell Signaling, 2820), 1:250, (Origene, CF809319); RNF2/RING1B (Cell Signaling, 5694), 1:250, (Abcam, ab101273), 1:500; STING (Cell Signaling, 13647), 1:1000; 1:500; Actin (Cell Signaling, 4970), 1:2000. Corresponding HRP-conjugated secondary antibodies (mouse, Cell Signaling, 7076 and rabbit, Cell Signaling, 7074) were added and band intensities were visualized using the ChemiDoc MP Imaging system and Image Lab software (Bio-Rad).

#### TCGA-UM expression and patients survival data

Expression datasets from the TCGA-UM cohort were accessed through cbioportal (https://www.cbioportal.org/study/summary?id=uvm_tcga)^91,92^ and survival and metastasis datasets were obtained from Robertson et al.^2^ Overall survival of patients in the dataset with high and low *STING* (*TMEM173*), *JARID2*, *RING1, ‘H2AK119Ub targets’* or *‘PRT-geneset’*; full list shown in (Supplementary Data File 1) expression were compared using the log-rank test. For transcript levels of individual genes, the z-normalized expression data from the dataset was used, and for genesets, the average expression of all genes was used. Tumors were labeled as *BAP1*-diploid and *BAP1*-deleted based on *BAP1* inferred copy number (cutoff value 0.2). Copy number and gene expression levels of *BAP1*, *RING1*, *RNF2* and *JARID2* were queried and plotted using cbioportal (Supplementary Fig. 6e).

### Reporting summary

Further information on research design is available in the Nature Research Reporting Summary linked to this article.

## Supplementary information

Supplementary Information

Description of Additional Supplementary Files

Supplementary Dataset 1

Supplementary Dataset 2

Supplementary Dataset 3

Supplementary Dataset 4

Supplementary Dataset 5

Supplementary Dataset 6

Supplementary Dataset 7

Supplementary Dataset 8

Supplementary Dataset 9

Reporting Summary

## Data Availability

The single cell RNA sequencing data generated in this study have been deposited in the NCBI’s Gene Expression Omnibus database under accession code GSE160883. The processed single cell data and example notebooks are available at https://github.com/LaughneyLab/uveal-melanoma. The RNA and CUT&RUN sequencing data generated in this study have been deposited in the NCBI’s Gene Expression Omnibus database under accession code GSE181600. The experimental data generated in this study (in Figs. 4b, 5c, d, f, 6a, b, d, 8a, b, and supplementary figures 6b, 8b, d, g–i, 9c–f, 10c, 11a, b) are provided in the Source Data file. Source data is provided with this paper. The following databases/datasets have been used in this study, KEGG database: https://www.genome.jp/kegg/; The validation single cell data cohort from Durante et. al^24^ was accessed from NCBI’s Gene Expression Omnibus database under accession code GSE139829. A ranked list of DEG and complete GSEA results per tumor archetype and cells analyzed in this manuscript are provided in Supplementary Data Files 2, 4-6. A custom GSEA annotation file, assembled to query cell types and pathways related to UM, PRC1/2 transcriptional signature and aneuploidy, as well as hallmark genesets is provided in Supplementary Data File 3. Source data are provided with this paper.

## Source data

Source Data
