# Supplementary material for: Loss of polycomb repressive complex 1 activity and chromosomal instability drive uveal melanoma progression
Source: Nat Commun. 2021 Sep 13;12:5402. doi: 10.1038/s41467-021-25529-z (PMC8438051; doi:10.1038/s41467-021-25529-z)
Supplement: Supplementary file 1 — Supplementary Information [file 41467_2021_25529_MOESM1_ESM.pdf]

Supplementary Information

Supplementary Table 1. Patients attributes and specimens' clinical and genetic profiles.

Patient	Gender, age (yrs)	Largest basal diameter x height (mm)	GEP	UM hotspot mutations	*BAP1* mutations	Copy number alterations	Metastasis (onset in months)	Vital status (follow up in months)
**UM1**	Male, 62	14.5 x 14.5	2	GNA11 R183H	R146T	NA	None	Alive (7)
**UM2**	Male, 60	15.3 x 12	2	GNA11.Q209L	None	3 loss	Liver, mediastinal nodes (26)	Deceased (37)
**UM3**	Female, 61	19 x 9	1B	GNAQ.Q209R, SF3B1.R625H	None	6q loss, 11 q loss	None	Alive (43)
**UM4**	Male, 72	22 x 4	2	GNA11 Q209L, TGFBR2	None	3 loss, 8q gain	None	Alive (27)
**UM5**	Male, 78	20 x 15	2	EIF1AX W70C, PARP1 I019V	F170C X86_splice	3 loss	None	Alive (39)
**UM6**	Female, 61	10 x 11	1A	GNA11 Q209L	K240*	3 loss, 8q gain, 16q loss, 17p loss and X loss	Liver and lungs (11)	Deceased (17)

Supplementary Figure 1: The single cell transcriptional landscape of human UM

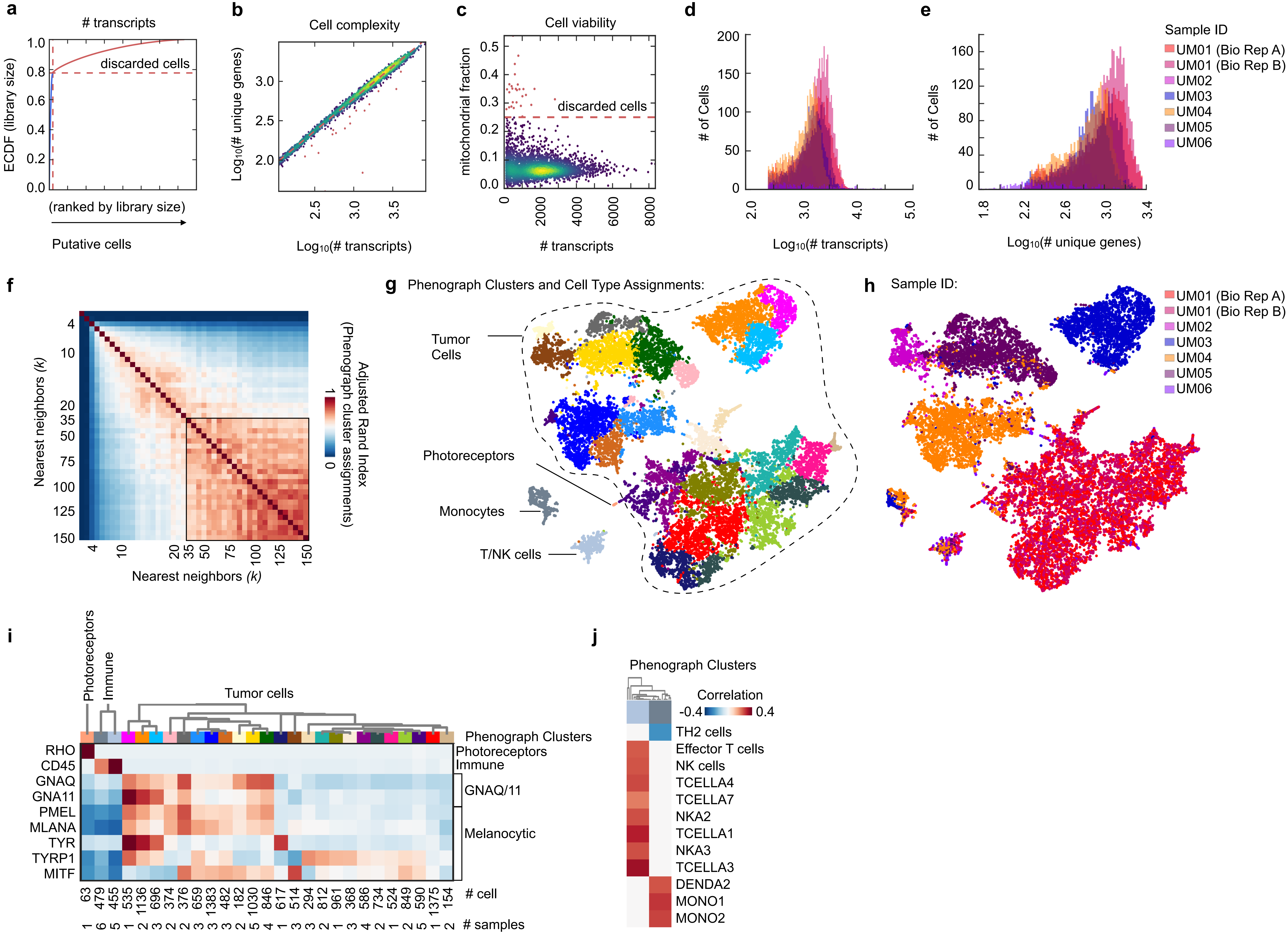

Supplementary Figure 1. The single cell transcriptional landscape of human uveal melanoma.

Cells were filtered based on (a) cumulative number of transcript counts, (b) cell complexity and (c) fraction of mitochondrial mRNA content detected per cell as described in the Methods; shown here for one representative patient sample. Excluded cells are labeled in red. Histograms showing the distribution of (d) total number of transcripts detected per cell and (e) number of unique genes detected per cell in retained cells colored by sample. Note: two independent libraries of single cells were isolated and profiled from patient UM01 (Bio Rep A & B), to assess library-specific batch effects.

(f) Robustness of Phenograph cluster assignments to nearest neighbor *k* was achieved by evaluating the adjusted rand index between Phenograph cluster assignments made using all pairwise values of nearest neighbors, *k*. For each pairwise comparison, we compute the ARI across all cells; visualized on a heatmap. Phenograph clustering was performed with $k = 35$ because this was the minimum value of *k* for which categorical assignments were stable (ARI > 0.75).

(g-h) t-SNE projection of the complete atlas of all patient cells recovered from primary UM colored by (g) Phenograph cluster assignment and (h) sample annotated in (*e*).

(i) Clustered heatmap of the average impute expression per Phenograph cluster of distinguishing markers, standardized by z-scores. Rows represent genes and columns are colored by Phenograph cluster as in (*g*). The number of cells and patients detected per Phenograph cluster are annotated below each column.

(j) Pearson correlations between Phenograph cluster centroids and bulk mRNA profiles from purified immune subpopulations [1, 2] (detailed in Methods). Correlations are whited out if $p > 0.01$ for the Pearson test for non-correlation. Columns (Phenograph clusters) are excluded if no significant positive correlation is observed between the Phenograph cluster and any bulk immune signatures.

Supplementary Figure 2: Large scale CNV events identified from scRNA-seq

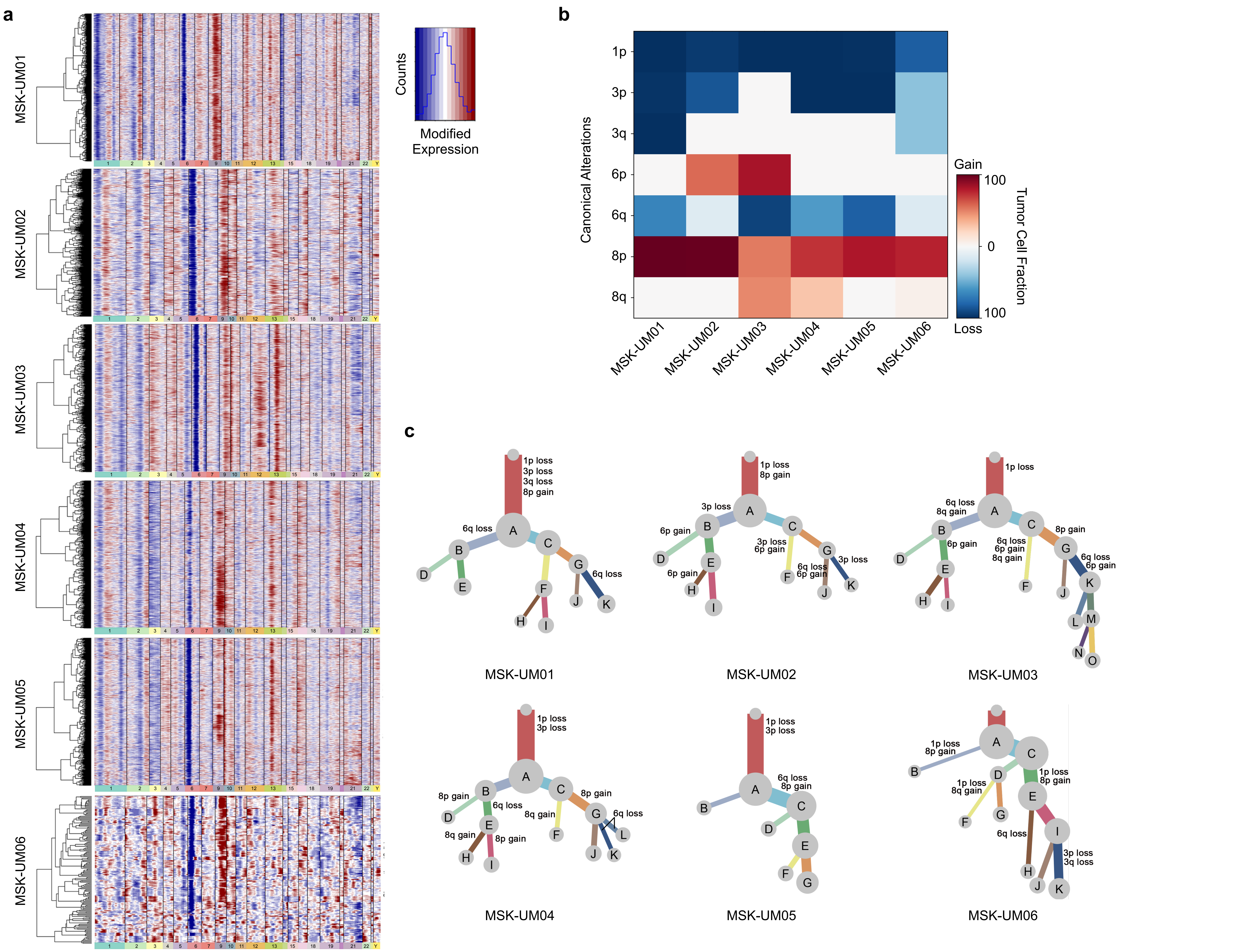

Supplementary Figure 2. Large scale CNV events identified from scRNA-seq.

(a) Heatmap of the modified gene expression computed by inferCNV (methods) from single cell RNAseq data ordered by chromosome region. Columns are genes ordered by chromosome region and rows represents cells clustered by the Ward distance metric. (b) Cellular frequency of canonical UM alterations detected per patient by inferCNV using the three state Hidden Markov Model (Methods). Losses are visualized in blue, gains are visualized in red, and the color saturation shows the percentage of cells predicted to contain the given alteration in each patient tumor. (c) Phylogenetic trees of the intra-patient subclones predicted by inferCNV using a hamming distance clustering method. Phylogeny shown per patient for subclones detected in >5% of each tumor. Canonical UM alterations are labeled. Branch width and node size are proportional to the fraction of each subclone detected per patient tumor.

Supplementary Figure 3: Clinical prognostication of individual tumor cells

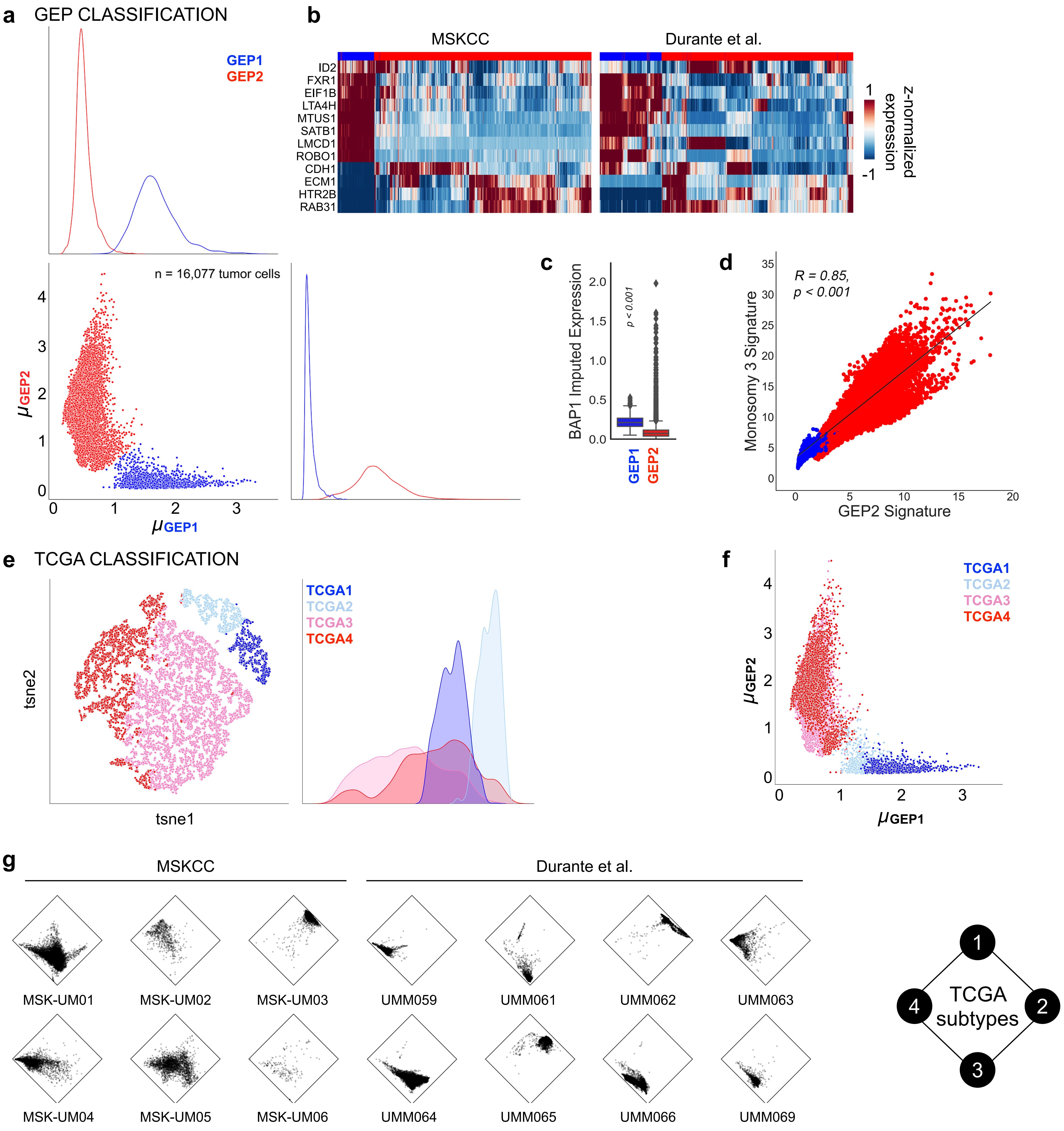

Supplementary Figure 3. Clinical prognostication of individual tumor cells.

(a) Individual tumor cells were assigned to GEP1 (blue) vs. GEP2 (red) clinical prognostic groups according to their average expression of the GEP prognostic gene signatures visualize on scatter plot, by fitting a two-component Bayesian Gaussian Mixture Model to this distribution, as described in the Methods. Each dot represents a tumor cell colored by its GEP assignment.

(b) Clustered heatmap of imputed expression of GEP prognostic genes, z-normalized across cells, in primary UMs from our cohort (MSKCC) and in an independent cohort (Durante et al.). Each column represents a cell colored by prognostic assignment GEP1 (n = 2304, blue) vs. GEP2 (n = 13,773, red). P = 0. Statistical significance tested using two-sided Mann-Whitney U rank test.

(c) Boxplots representing *BAP1* imputed expression across individual tumor cells assigned to GEP1 vs. GEP2 prognostic groups (center line, median; box limits, upper and lower quartiles; whiskers, 1.5x interquartile range; points, outlier cells).

(d) Correlation between the average imputed expression of the GEP2 and Monosomy 3 signatures (annotated in **Supplementary Data File 1**) per cell, colored by its assignment to GEP1 (blue) or GEP2 (red). Black line indicates linear fit (Pearson correlation $R = 0.86$ and $p < 0.001$). P = 0. Statistical significance tested using two-tailed Pearson correlation.

(e) Individual tumor cells were likewise assigned to TCGA molecular subtypes according to their average expression of characteristic genes (annotated in **Supplementary Data File 1**) by fitting a four-component Bayesian Gaussian Mixture Model to this distribution, as described in the Methods. Each dot represents a tumor cell colored by its TCGA molecular subtype. Average expression of characteristic genes for each subtype is projected onto two dimensions using t-distributed stochastic neighbor embedding.

(f) TCGA assignments are also visualized on a two-dimensional scatterplot reflecting average expression of the GEP prognostic gene signatures. High concordance is observed between good and poor prognostic classes, regardless of risk stratification.

(g) Diamond plots reflecting the relative expression of TCGA molecular subtypes signature genes. For each patient, the mean imputed expression of characteristic genes (annotated in **Supplementary Data File 1**) is calculated per cell and normalized across subtypes, such that the sum of expression is 1 per cell. Normalized mean imputed expression values are mapped to two dimensions by assigning the difference between mean expression of TCGA Subtype 2 and Subtype 4 to x-axis values and the difference between mean expression of TCGA Subtype 1 and Subtype 3 to the y-axis values on the diamond plot. In this way, the origin represents a cell that has equal contributions from all four TCGA molecular subtypes and each corner of the diamond represents a cell that associates exclusively with one molecular subtype. This leads to cells that are mostly TCGA molecular subtypes 1 and 2 (good prognosis), clustering near the top right edge of the diamond and cells that are mostly subtype 3 and 4 (poor prognosis) clustering near the bottom left edge of the diamond.

Supplementary Figure 4: Tumor archetypal analyses reveals extreme phenotypic states defined by chromatin modifications, aneuploidy, and inflammatory response genes.

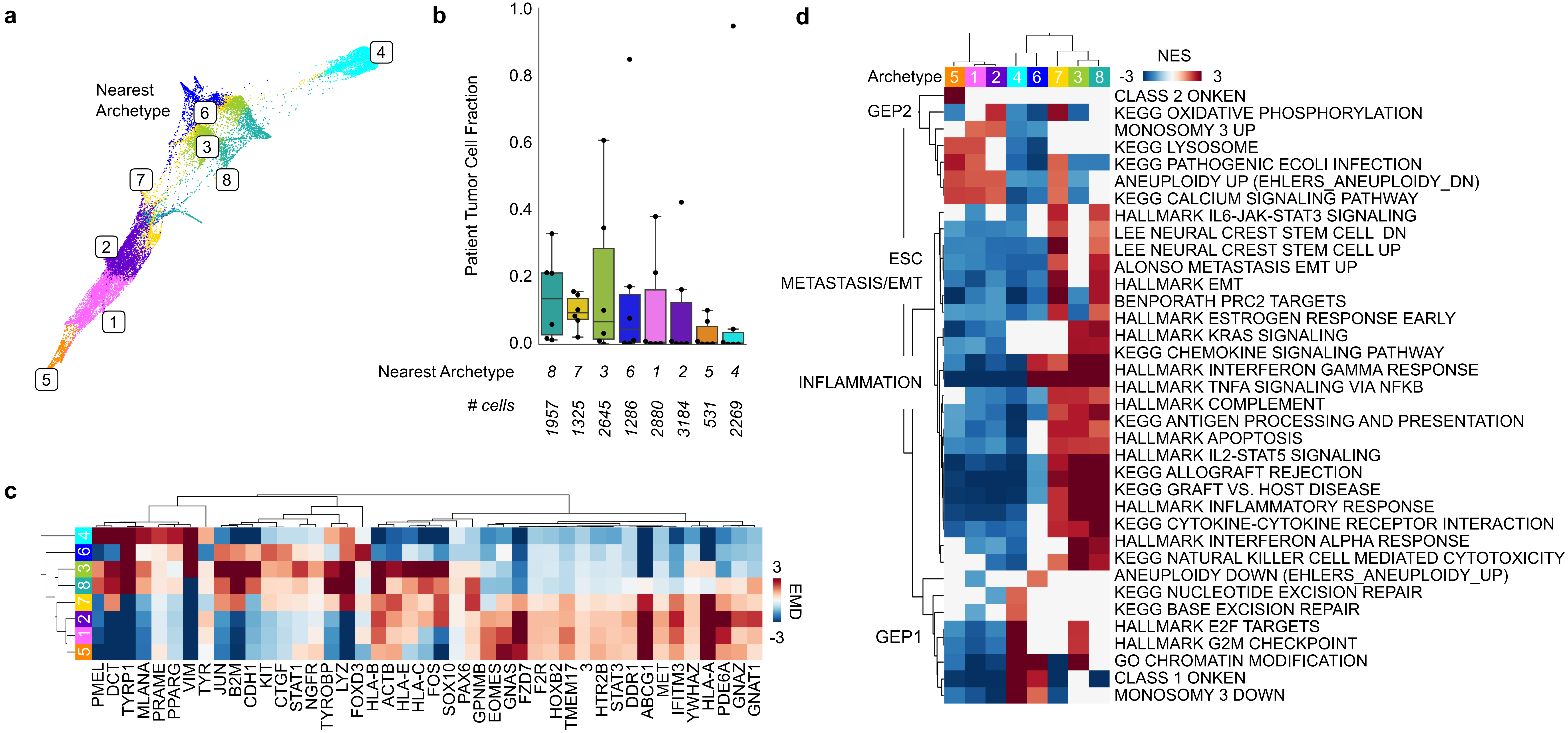

Supplementary Figure 4. Archetypal analysis of tumor cells reveals extreme phenotypic states defined by chromatin modifications, aneuploidy, and inflammatory response genes.

(a) Force-directed layout of all patient tumor cells labeled and colored by nearest archetype (see *Methods*); archetypes represent extreme phenotypic states.

(b) Fraction of patient tumor cells assigned to the nearest archetype (center line, median; box limits, upper and lower quartiles; whiskers, 1.5x interquartile range; points, outliers); n = 6 samples.

(c) EMD scores for a subset of differentially expressed genes per archetype (columns), including genes involved in melanogenesis, inflammation, antigen presentation and migration. EMD scores for all differentially expressed genes are listed in **Supplementary Data File 2**.

(d) Clustered heatmap of gene signatures differentially expressed by one or more tumor archetypes. The normalized enrichment score (NES) is shown for signatures with $\text{abs(NES)} > 1.5$ $p_{adj} < 0.25$ and $p < 0.05$; column colors (top) correspond to annotated tumor archetypes. Signatures not meeting these criteria are whited out. Complete GSEA results per tumor archetype, including nominal p-values, are listed in **Supplementary Data File 4**.

Supplementary Figure 5: Stratification of UM cell lines

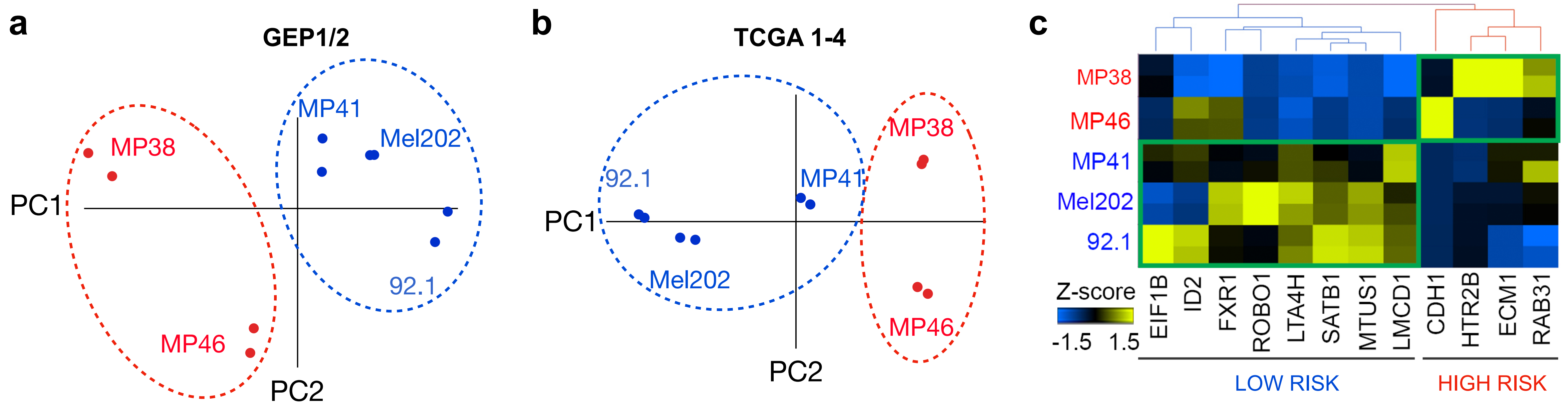

Supplementary Figure 5. Stratification of UM cells

(a) Principal component analysis of UM cells based on expression of the clinically-validated, discriminant 12 gene set (**Supplementary Data File 1**). X and y axis showing principal component (PC) 1 and 2.

(b) Principal component analysis of UM cells based on expression of the TCGA discriminant 80 gene set (**Supplementary Data File 1**). X and y axis showing principal component (PC) 1 and 2.

For (a) and (b), biological duplicates are shown for each cell line.

(c) Unbiased hierarchical clustering of UM cell lines, in biological duplicates, based on normalized FPKM values of the GEP1 and 2, 12-discriminant geneset [3]. High (red) and low risk (blue) transcriptional phenotype.

Supplementary Figure 6: Differential expression of PRC1 components in low-risk and high-risk UM

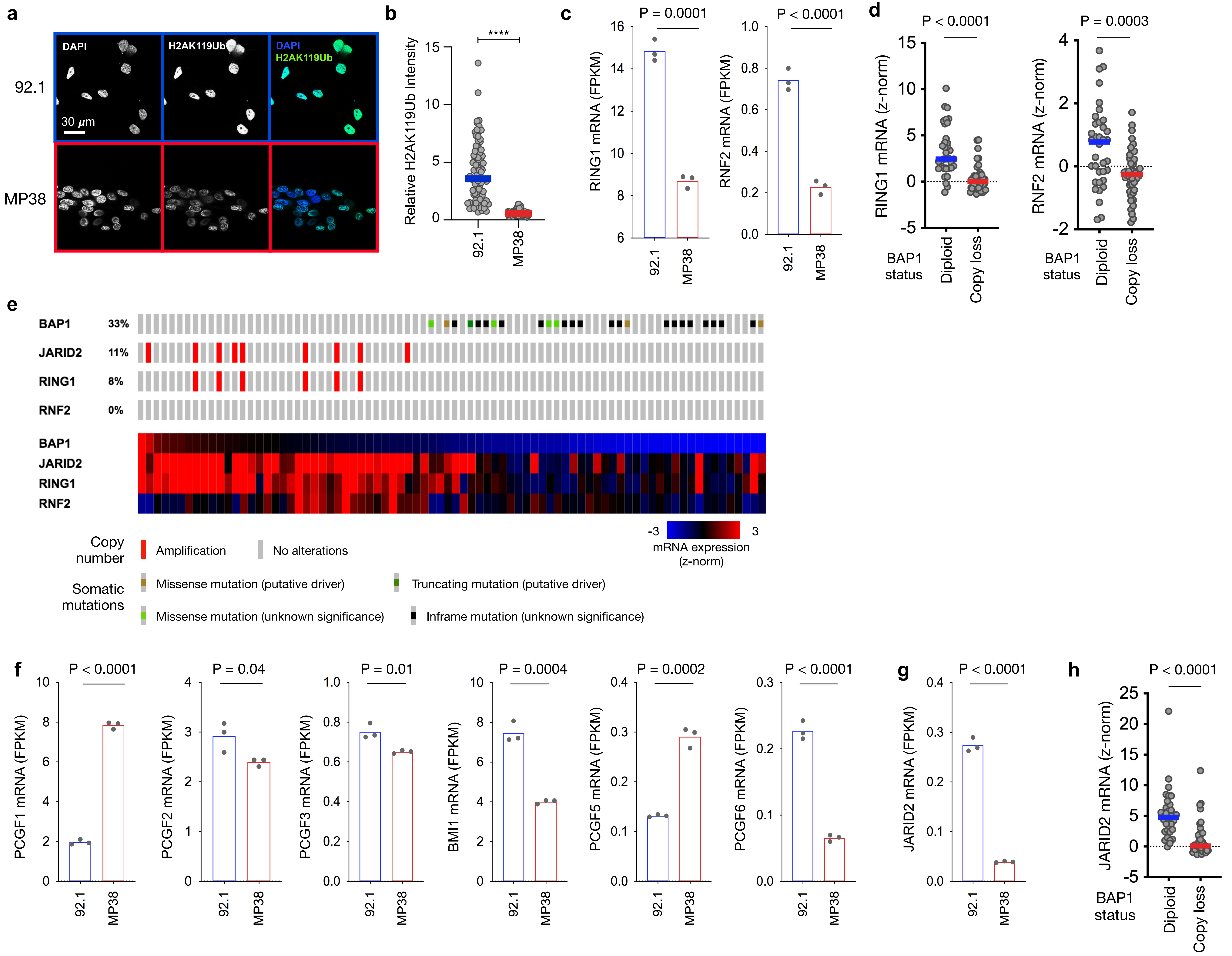

Supplementary Figure 6. Differential expression of core PRC1 components in low-risk and high-risk UM

(a) Representative immunofluorescence staining of H2AK119Ub (green) and DAPI (blue) in low risk, 92.1 and high risk, MP38 UM cells. Representative images from biological triplicates.

(b) Quantification of H2AK119Ub relative to DAPI in low risk, 92.1 and high risk, MP38 UM cells. Data representative of biological triplicates. Bar, mean; circles, individual values. Source data are provided as a Source Data file.

(c) Transcript levels of core PRC1 ligases, *RING1* and *RNF2* in high-risk MP38, and low risk 92.1 UM cells. Bar, mean; circles, individual FPKM values (biological triplicates). P (RING1) = 5.9×10^{-5}.

(d) Transcript levels of core PRC1 ligases, *RING1* and *RNF2* in 80 primary UMs with diploid *BAP1,* or with *BAP1* genomic copy loss. Source TCGA-UM. Bar, median; circles, z-normalized values from individual tumors. P (RING1) = 1.0×10^{-8}.

(e) Oncoprint of 80 TCGA-UM tumors showing mutual exclusivity between *BAP1* mutations and copy number amplification in *JARID2* and *RING1*. *Lower panel*, heatmap of transcript levels showing high concordance between mRNA levels of *BAP1*, *JARID2*, *RING1* and *RNF2*. Samples arranged based on *BAP1* mRNA levels. Source: TCGA-UM cohort. Data visualized using cbioportal software [4, 5].

(f) Transcript levels of accessory PRC1 components *PCGF1-6* in high-risk MP38, and low risk 92.1 UM cells. Bar, mean; circles, individual FPKM values (biological triplicates). P (PCGF1) = 1.2×10^{-6}; p (PCGF6) = 4.7×10^{-5}.

(g) Transcript levels of *JARID2* in high-risk MP38, and low risk 92.1 UM cells. Bar, mean; circles, individual FPKM values (biological triplicates). P = 9.2 x 10^{-6}.

(h) Transcript levels of *JARID2* in 80 primary UMs with diploid *BAP1,* or with *BAP1* genomic copy loss. Source TCGA-UM. Bar, median; circles, z-normalized values from individual tumors. P = 5.2 x 10^{-7}.

Statistical significance tested using two-sided unpaired Student's t-test (b-d, f-h).

Supplementary Figure 7: Loss of PRC1 in high-risk UM

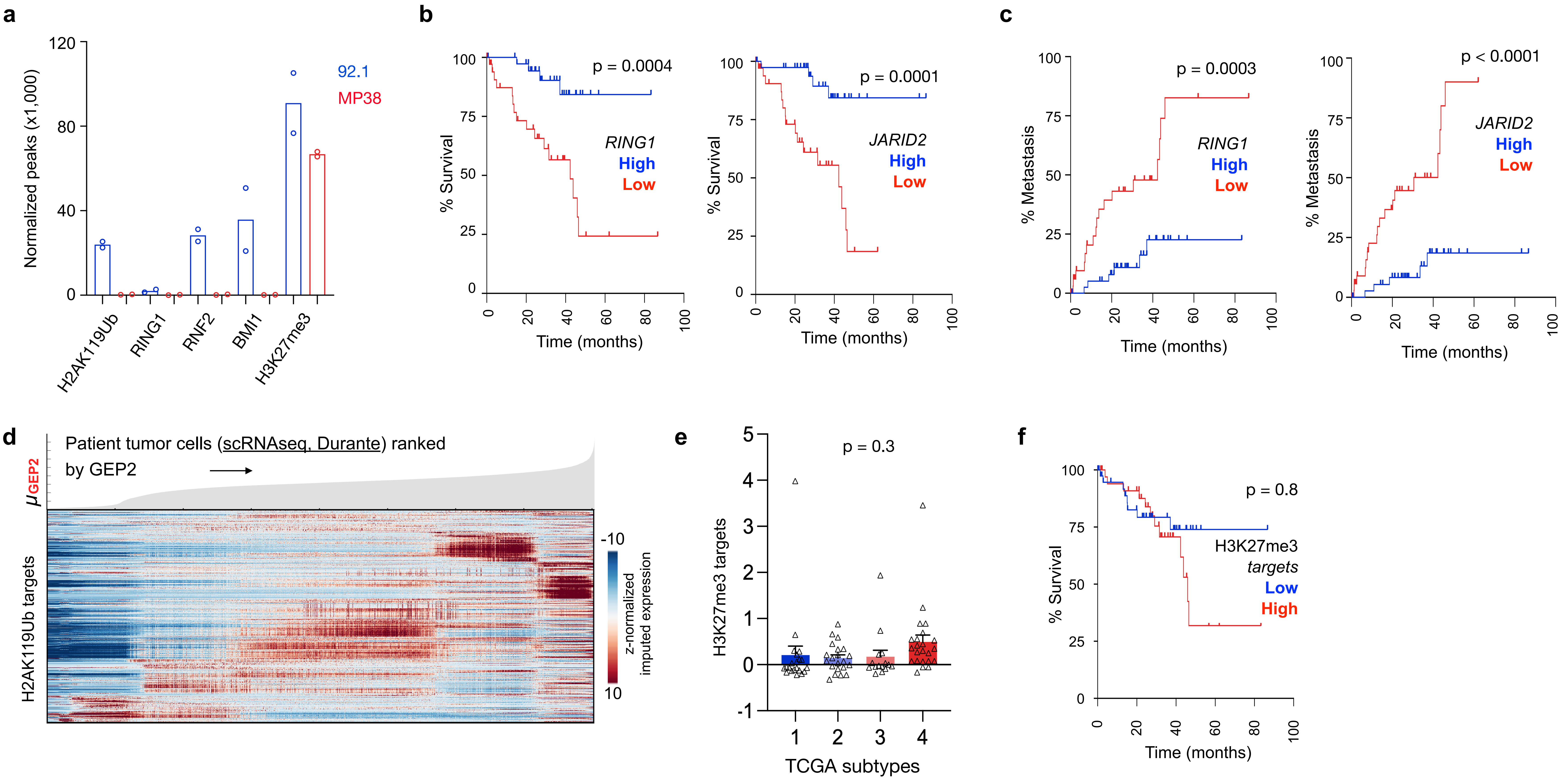

Supplementary Figure 7. Loss of PRC1 in high risk UM.

(a) Genomic peaks from CUT&RUN of H2AK119Ub, RING1, RNF2, BMI1 and H3K27me3 normalized to IgG in 92.1 (blue) and MP38 (red). Data representative of biological duplicates.

(b) Overall survival of (n = 80) TCGA-UM patients with primary tumors stratified by expression of high (top 50th percentile, n=40) and low (bottom 50th percentile, n = 40) *JARID2* and *RING1*.

(c) UM-related metastasis of (n = 80) TCGA-UM patients with primary tumors stratified by expression of high (top 50th percentile, n=40) and low (bottom 50th percentile, n = 40) expression of *RING1* and *JARID2*. P = 3.0×10^{-5}.

(d) Expression of '*H2AK119Ub targets*' geneset (see Methods for gene signature annotated in **Supplementary Data File 1**) for all individual tumor cells obtained from Durante et al. [6], ranked by average imputed expression of the GEP2 gene signature (gene signatures annotated in **Supplementary Data File 1**) in ascending order from left to right. For each gene, imputed expression was z-normalized across all cells and smoothed using a 20-cell moving average window. *Top,* filled area plot showing average expression of the GEP2 signature across ranked tumor cells.

(e) Expression of H3K27me3 target genes across the 4 molecular TCGA subtypes.

(f) Overall survival of (n = 80) TCGA-UM patients with primary tumors stratified by high (top 50th percentile, n=40) and low (bottom 50th percentile, n = 40) expression of the 'H3K27me3 targets' geneset.

Statistical significance tested using two-sided log-rank test (b), (c) and (f) and one-way ANOVA (e).

Supplementary Figure 8: PRC1 inhibition induces H2AK119 de-ubiquitination and morphological changes in low risk, but not high risk UM

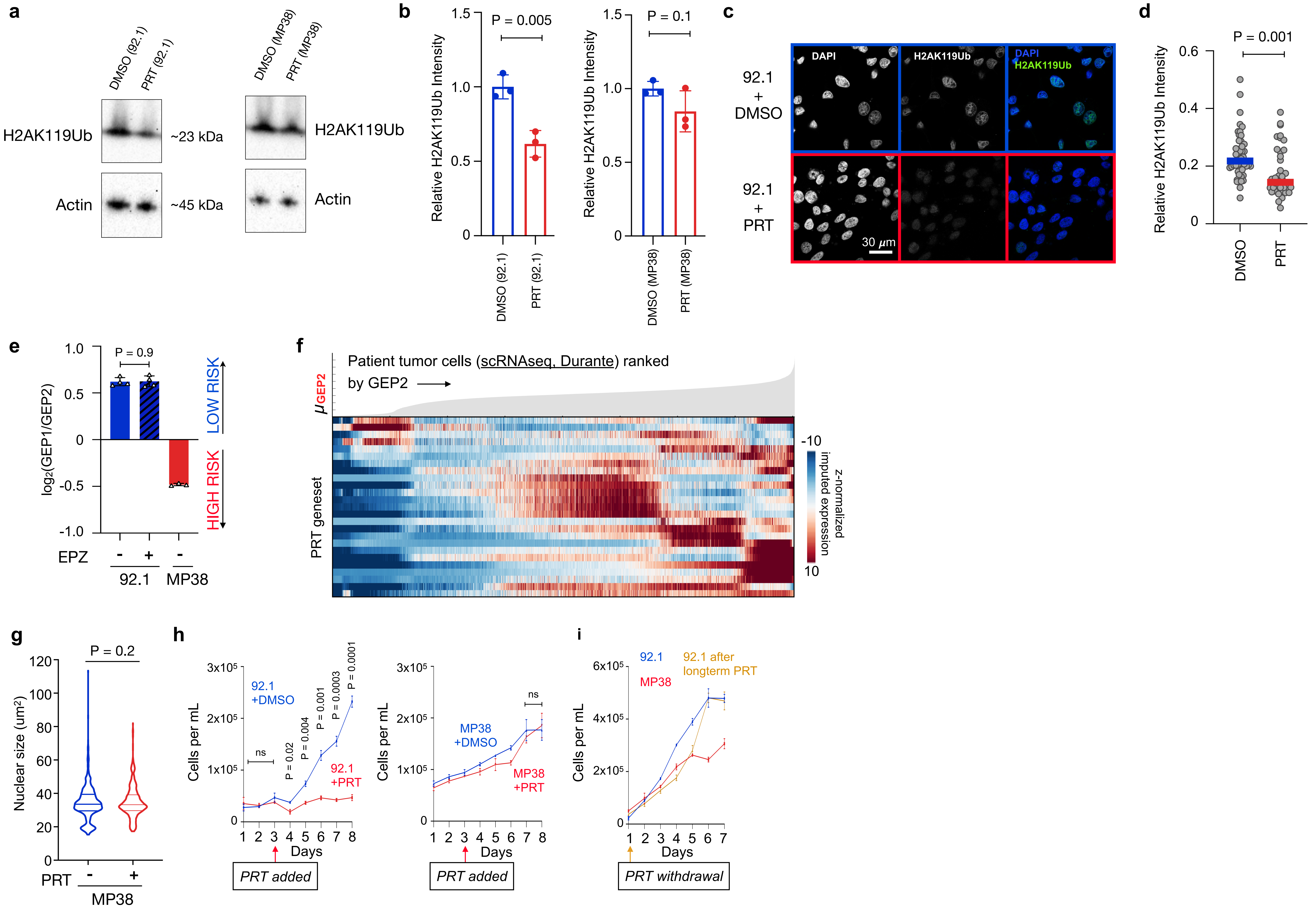

Supplementary Figure 8. PRC1 inhibition induces H2AK119 de-ubiquitination and morphological changes in low risk, but not high risk, UM

(a) Western blot of H2AK119Ub relative to Actin in low risk, 92.1 and high risk, MP38 UM cells upon PRT or DMSO treatment after 2 h. Data representative of biological triplicates.

(b) Quantification of H2AK119Ub normalized to Actin. Bars, standard deviation; circles, biological triplicates. Source data are provided as a Source Data file.

(c) Representative immunofluorescence staining of H2AK119Ub (green) and DAPI (blue) in DMSO or PRT-treated 92.1 cells after 2h. Representative images from biological triplicates.

(d) Quantification of H2AK119Ub signal intensity relative to DAPI. In DMSO or PRT-treated 92.1 cells. Experimental triplicates. Source data are provided as a Source Data file.

(e) Ratio of GEP1/GEP2 average gene signature expression (gene signatures annotated in **Supplementary Data File 1**) in low-risk UM cells (92.1) upon 24h DMSO or EPZ-treatment, and high-risk UM cells (MP38) upon 24h DMSO-treatment. Error bars, standard error of the mean. Biological triplicates.

(f) Expression of '*PRT-geneset*' (see Methods for gene signature annotated in **Supplementary Data File 1**) for all individual tumor cells obtained from Durante et al. [6], ranked by average imputed expression of the GEP2 gene signature (gene signatures annotated in **Supplementary Data File 1**) in ascending order from left to right. For each gene, imputed expression was z-normalized across all

cells and smoothed using a 20-cell moving average window. *Top,* filled area plot showing average expression of the GEP2 signature across ranked tumor cells.

(g) Violin plots showing the distribution of nuclei size in high-risk UM cells (MP38) upon PRT (n = 510) and DMSO (n = 523) treatment for 48 hours. Lines distinguish interquartile ranges; width reflects observed nuclei number. Biological triplicates. Source data are provided as a Source Data file.

(h) Growth of low risk, 92.1 UM cells (*left*) and high risk, MP38 UM cells (*right*) with DMSO (blue) or PRT (red) treatment. Arrow indicates the day when PRT was added. Error bars, standard error of the mean. Biological triplicates. Source data are provided as a Source Data file.

(i) Growth of high risk, MP38 UM cells (red), and low risk 92.1 UM cells after long-term treatment with DMSO (blue) or PRT (yellow). Error bars, standard error of the mean across biological triplicates. Source data are provided as a Source Data file.

Statistical significance tested using two-sided unpaired Student's t test (b), (d), (e), (g) and (h).

Supplementary Figure 9: CIN in high-risk UM

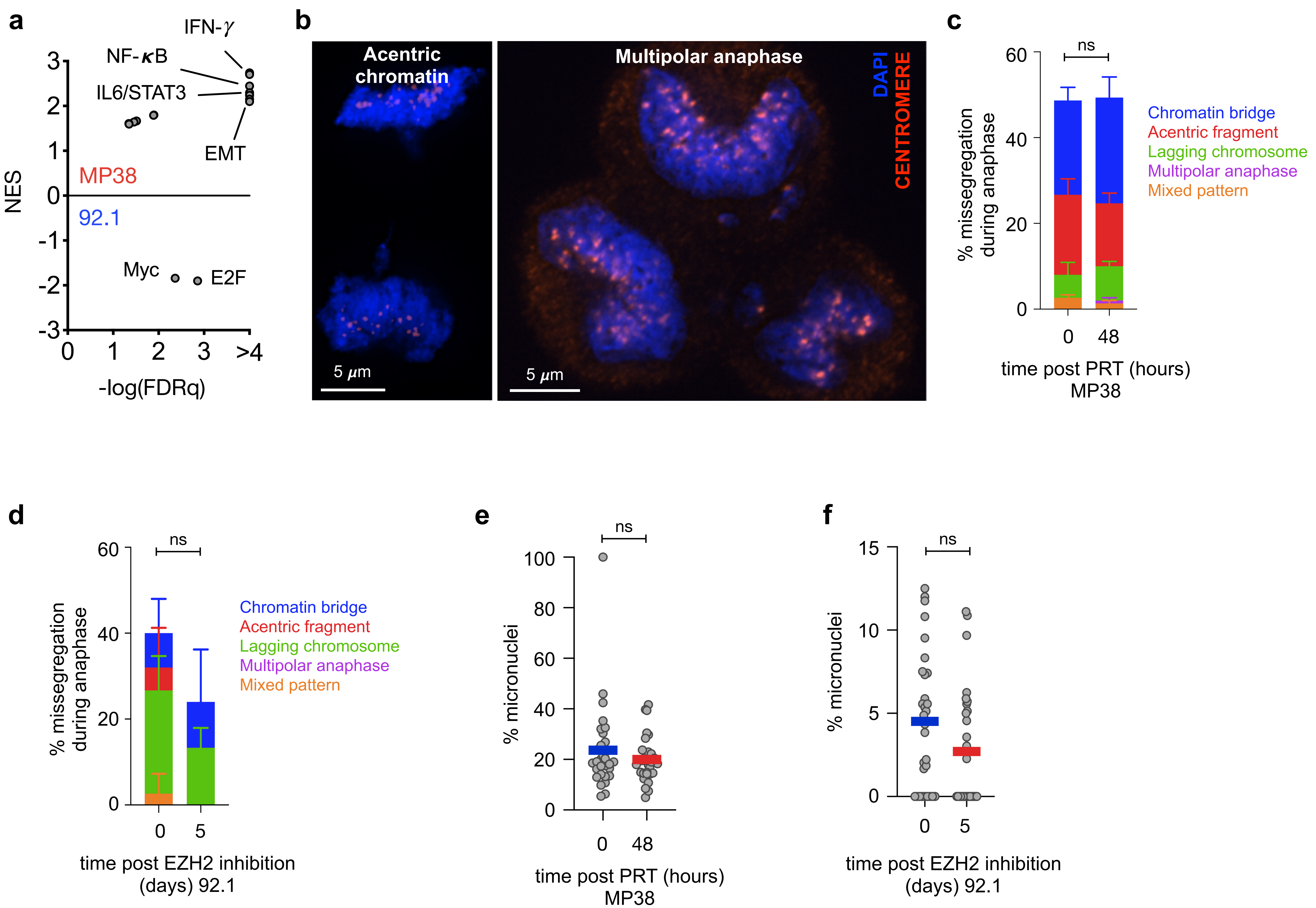

Supplementary Figure 9. CIN in high-risk UM

(a) Top differentially expressed pathways in high risk MP38 vs low risk 92.1 UM cells. Normalized enrichment score (NES) for selected genesets shown as a function of significance $-\log_{10}$(FDRq). Biological duplicates. An unfiltered list of all gene signatures is provided in **Supplementary Data File 6**.

(b) Examples of anaphase cells stained for DAPI (blue) and centromeres (red) demonstrating different patterns of chromosome segregation errors. Additional patterns are shown in **Fig. 5b**. Representative images from biological triplicates.

(c) Abundance of chromosome segregation error patterns during anaphase in high-risk MP38 UM cells upon DMSO or PRT-treatment for 48h across biological triplicates. Source data are provided as a Source Data file.

(d) Abundance of chromosome segregation error patterns during anaphase in low-risk 92.1 UM cells upon DMSO or GSK126 -treatment for 48h across biological triplicates. Source data are provided as a Source Data file.

(e) Micronuclei frequency in high-risk MP38 UM cells upon PRT treatment for 48h. Source data are provided as a Source Data file.

(f) Micronuclei frequency in low-risk 92.1 UM cells upon DMSO or GSK126 -treatment for 48h. Source data are provided as a Source Data file.

Statistical significance tested using two-sided student *t*-test (c - f).

Supplementary Figure 10: Inflammation and elevated STING in high-risk UM

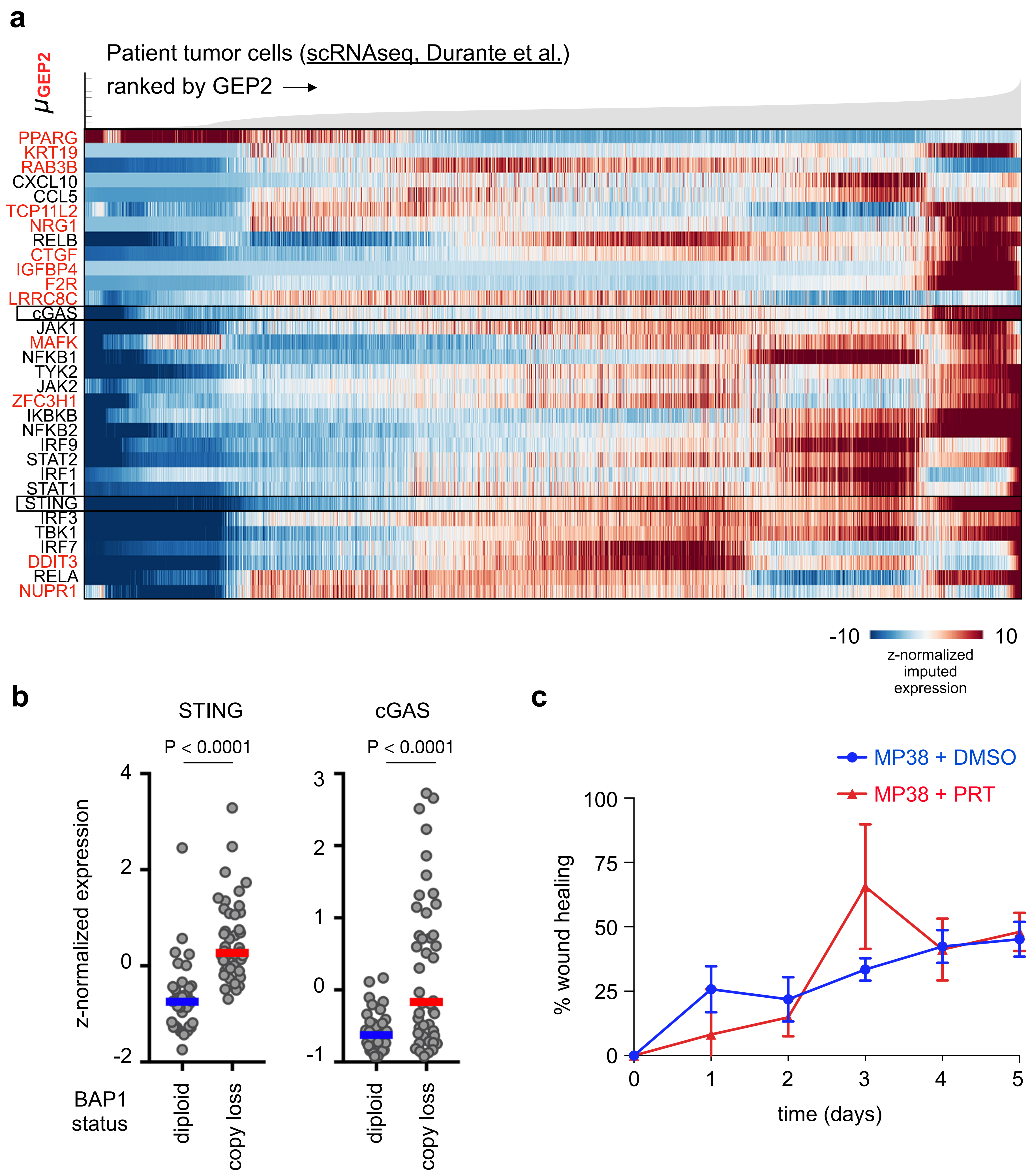

Supplementary Figure 10. Inflammation and elevated STING in high-risk UM

(a) Expression of key cytosolic nucleic acids sensors, intermediate signaling adapters, executioners, interferon stimulated genes (ISGs) and CIN-induced non-canonical NF-kB targets (*shown in red*) [7] for all individual patient tumor cells obtained from an independent single cell analysis of eight primary UM [6], ranked by average imputed expression of the GEP2 gene signature (gene signatures annotated in **Supplementary Data File 1**) in ascending order from left to right. For each gene, imputed expression was z-normalized across all cells and smoothed using a 20-cell moving average window. *Top,* filled area plot showing average expression of the GEP2 signature across ranked tumor cells.

(b) Transcript levels of *STING (TMEM173)* and *cGAS* in 80 primary UMs with diploid *BAP1,* or with *BAP1* genomic copy loss. Source TCGA-UM. Bar, median; circles, z-normalized values from individual tumors. Statistical significance tested using two-sided student *t*-test. P (STING) = 4.0×10^{-9}; p (cGAS) = 1.1×10^{-5}.

(c) Wound scratch assay to assess migratory potential of high risk MP38 UM cells upon treatment with DMSO or PRT. Experimental triplicates. Error bars, standard error of the mean. Source data are provided as a Source Data file.

Supplementary Figure 11: Pharmacologic modulation of CIN

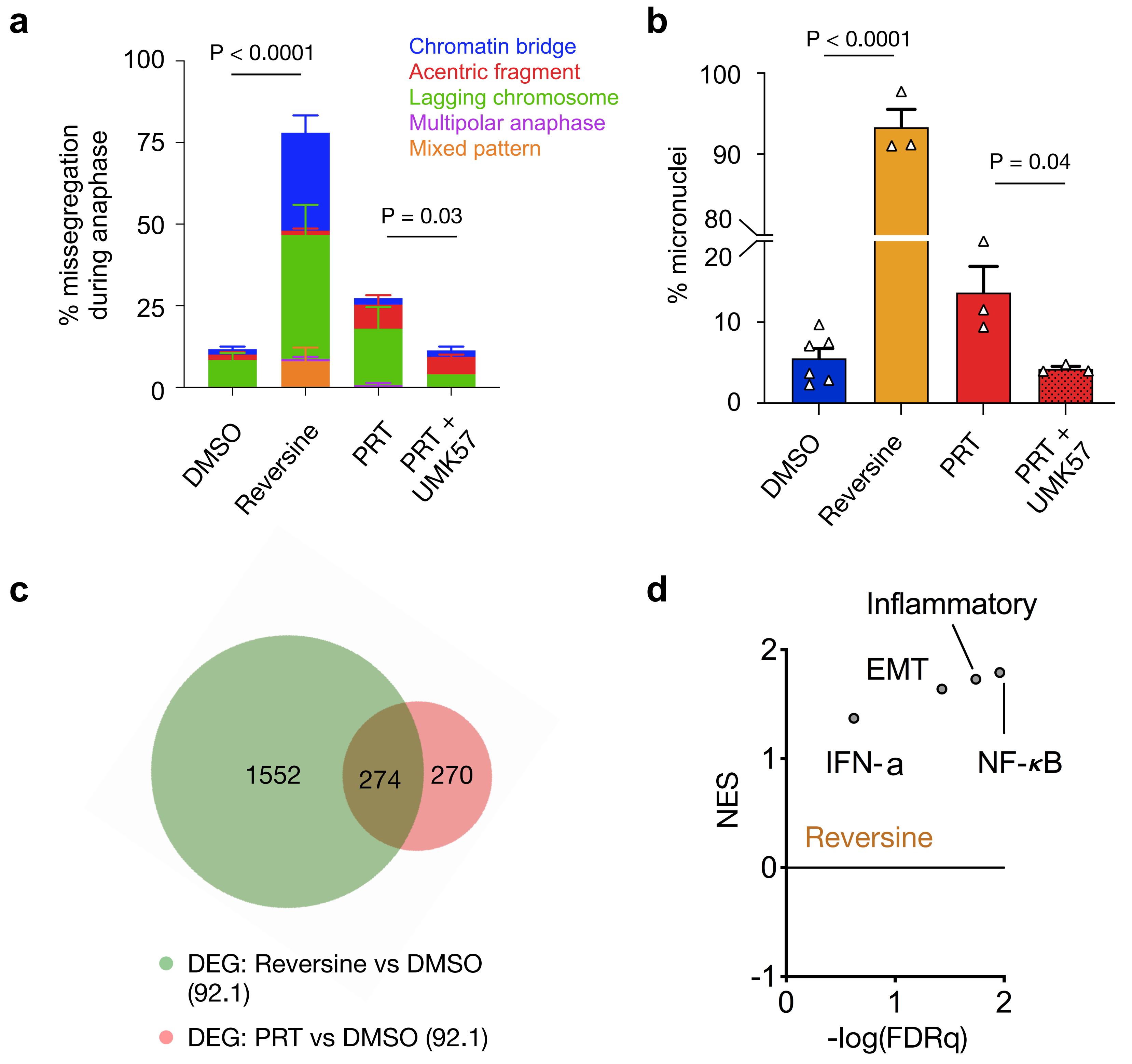

Supplementary Figure 11. Pharmacologic modulation of CIN

(a) Abundance of chromosome segregation error patterns during anaphase in low-risk 92.1 UM cells upon DMSO, reversine, PRT or PRT and UMK57 treatment for 48h. Stacked bars, mean of each missegregation pattern; error bars, standard error of the mean across experimental triplicates. $P = 4.7 \times 10^{-8}$. Source data are provided as a Source Data file.

(b) Micronuclei frequency in low risk 92.1 UM cells upon DMSO, reversine, PRT or PRT and UMK57 treatment for 48h. Bars, mean; error bars, standard error of the mean across experimental triplicates. $P = 2.2 \times 10^{-9}$. Source data are provided as a Source Data file.

(c) Venn diagram of DEG upon PRT (red) and reversine (green) treatment (92.1 cells).

(d) Differentially expressed pathways in low risk 92.1 UM cells upon reversine treatment. Normalized enrichment score (NES) for selected genesets shown as a function of significance $-\log_{10}$(FDRq). Biological triplicates. An unfiltered list of all gene signatures is provided in **Supplementary Data File 7**.

Statistical significance tested using two-sided unpaired Student's t-test (a) and (b).

References cited in Supplementary file

1. Novershtern N*, et al.* Densely interconnected transcriptional circuits control cell states in human hematopoiesis. *Cell* **144**, 296-309 (2011).

2. Jeffrey KL*, et al.* Positive regulation of immune cell function and inflammatory responses by phosphatase PAC-1. *Nat Immunol* **7**, 274-283 (2006).

3. Onken MD, Worley LA, Tuscan MD, Harbour JW. An accurate, clinically feasible multi-gene expression assay for predicting metastasis in uveal melanoma. *J Mol Diagn* **12**, 461-468 (2010).

4. Cerami E*, et al.* The cBio cancer genomics portal: an open platform for exploring multidimensional cancer genomics data. *Cancer Discov* **2**, 401-404 (2012).

5. Gao J*, et al.* Integrative analysis of complex cancer genomics and clinical profiles using the cBioPortal. *Sci Signal* **6**, pl1 (2013).

6. Durante MA*, et al.* Single-cell analysis reveals new evolutionary complexity in uveal melanoma. *Nat Commun* **11**, 496 (2020).

7. Bakhoum SF*, et al.* Chromosomal instability drives metastasis through a cytosolic DNA response. *Nature* **553**, 467-472 (2018).
