# Supplementary material for: Loss of polycomb repressive complex 1 activity and chromosomal instability drive uveal melanoma progression
Source: Nat Commun. 2021 Sep 13;12:5402. doi: 10.1038/s41467-021-25529-z (PMC8438051; doi:10.1038/s41467-021-25529-z)
Supplement: Supplementary file 2 — Description of Additional Supplementary Files [file 41467_2021_25529_MOESM2_ESM.docx]

**Description of Additional Supplementary Files**

**Title: Supplementary Data File 1.**

Description: Gene signatures plotted in data figures.

**Title: Supplementary Data File 2.**

Description: Differentially expressed genes per tumor archetype.

**Title: Supplementary Data File 3.**

Description: Pathways and genesets tested in GSEA (GMT file).

**Title: Supplementary Data File 4.**

Description: Complete GSEA results per tumor archetype.

**Title: Supplementary Data File 5.**

Description: Complete GSEA results for PRT versus DMSO treatment of low-risk 92.1 UM cells.

**Title: Supplementary Data File 6.**

Description: Complete GSEA results for high-risk MP38 versus low-risk 92.1 UM cells.

**Title: Supplementary Data File 7.**

Description: Complete GSEA results for low-risk 92.1 UM cells upon reversine treatment.

**Title: Supplementary Data File 8.**

Description: NanoString custom gene panel.

**Title: Supplementary Data File 9.**

Description: ImageJ Script used for immunofluorescence signal quantification (IJM file).
