# Supplementary material for: Loss of polycomb repressive complex 1 activity and chromosomal instability drive uveal melanoma progression
Source: Nat Commun. 2021 Sep 13;12:5402. doi: 10.1038/s41467-021-25529-z (PMC8438051; doi:10.1038/s41467-021-25529-z)
Supplement: Supplementary file 12 — Reporting Summary [file 41467_2021_25529_MOESM12_ESM.pdf]

nature research

Corresponding author(s): Ashley Laughney, PhD, Samuel Bakhoum, MD, PhD, Paul Mischel, MD and Mathieu Bakhoum, MD, PhD

Last updated by author(s): 07/16/2021



# Reporting Summary

Nature Research wishes to improve the reproducibility of the work that we publish. This form provides structure for consistency and transparency in reporting. For further information on Nature Research policies, see Authors & Referees and the Editorial Policy Checklist .

## Statistics

For all statistical analyses, confirm that the following items are present in the figure legend, table legend, main text, or Methods section.

| n/a | Confirmed | |
|---|---|---|
| ☐ | ☒ | The exact sample size (*n*) for each experimental group/condition, given as a discrete number and unit of measurement |
| ☐ | ☒ | A statement on whether measurements were taken from distinct samples or whether the same sample was measured repeatedly |
| ☐ | ☒ | The statistical test(s) used AND whether they are one- or two-sided<br>*Only common tests should be described solely by name; describe more complex techniques in the Methods section.* |
| ☒ | ☐ | A description of all covariates tested |
| ☐ | ☒ | A description of any assumptions or corrections, such as tests of normality and adjustment for multiple comparisons |
| ☐ | ☒ | A full description of the statistical parameters including central tendency (e.g. means) or other basic estimates (e.g. regression coefficient) AND variation (e.g. standard deviation) or associated estimates of uncertainty (e.g. confidence intervals) |
| ☐ | ☒ | For null hypothesis testing, the test statistic (e.g. *F*, *t*, *r*) with confidence intervals, effect sizes, degrees of freedom and *P* value noted<br>*Give P values as exact values whenever suitable.* |
| ☐ | ☒ | For Bayesian analysis, information on the choice of priors and Markov chain Monte Carlo settings |
| ☒ | ☐ | For hierarchical and complex designs, identification of the appropriate level for tests and full reporting of outcomes |
| ☐ | ☒ | Estimates of effect sizes (e.g. Cohen's *d*, Pearson's *r*), indicating how they were calculated |

*Our web collection on statistics for biologists contains articles on many of the points above.*

## Software and code

Policy information about availability of computer code

Data collection

No Software was used

Data analysis

Partek flow software, version 10.0 (Partek) for bulk RNA sequencing and CUT&RUN analysis, Python 3.6.9 for single cell analysis.

Below are a list of all packages used adjustText v. 0.7.3 alabaster v. 0.7.9 anaconda-client v. 1.2.2 anndata v. 0.7.4 annoy v. 1.16.3 appdirs v. 1.4.3 apptools v. 4.4.0 argcomplete v. 1.0.0 argon2-cffi v. 20.1.0 arrow v. 0.7.0 asn1crypto v. 0.24.0 astroid v. 1.4.7 astunparse v. 1.6.3 attrdict v. 2.0.0 attrs v. 19.3.0 awscli v. 1.18.114 Babel v. 2.3.4 backcall v. 0.2.0 backports.shutil-get-terminal-size v. 1.0.0 bcrypt v. 3.1.6 beautifulsoup4 v. 4.7.1 bhtsne v. 0.1.9 biopython v. 1.73 bioservices v. 1.6.0 bitarray v. 0.8.1 blaze v. 0.10.1 bleach v. 3.1.5 bokeh v. 0.12.2 boto v. 2.42.0 boto3 v. 1.14.37 botocore v. 1.17.23 Bottleneck v. 1.2.1 CacheControl v. 0.12.0 cachetools v. 4.1.1 cairocffi v. 0.8.0 CairoSVG v. 2.3.0 certifi v. 2020.6.20 cffi v. 1.12.2 chardet v. 3.0.4 charlotte v. 0.1.0 chest v. 0.2.3 click v. 6.6 cloudpickle v. 1.3.0 clyent v. 1.2.1 cmake v. 3.18.0 cmocean v. 2 colorama v. 0.3.9 colorlog v. 4.0.2 colors.py v. 0.2.2 ConcurrentLogHandler v. 0.9.1 configobj v. 5.0.6 contextlib2 v. 0.5.3 coverage v. 4.5.3 cronex v. 0.1.0 cryptography v. 2.6.1 cssselect2 v. 0.2.1 cvxopt v. 1.1.8 cycler v. 0.10.0 Cython v. 0.29.21 cytoolz v. 0.8.0 dask v. 0.11.0 datashape v. 0.5.2 decorator v. 4.4.2 defusedxml v. 0.5.0 dill v. 0.2.9 distro-info v. 0.18ubuntu0.18.04.1 docutils v. 0.14 doubletdetection v. file:///opt/DoubletDetection easydev v. 0.9.37 easydict v. 1.9 ecdsa v. 0.13 echarts-python v. 0.1.3 entrypoints v. 0.3 et-xmlfile v. 1.0.1 ete3 v. 3.0.0b35 fa2 v. 0.2 fastcache v. 1.0.2 fastcluster v. 1.1.25 fcsparser v. 0.1.2 filelock v. 2.0.6 Flask v. 1.1.2 Flask-Cors v. 3.0.8 ftputil v. 3.3.1 future v. 0.16.0 gast v. 0.3.3 geode v. get-version v. 2.1 gevent v. 1.4.0 gipc v. 0.6.0 google-auth v. 1.19.2 google-auth-oauthlib v. 0.4.1 google-pasta v. 0.2.0 greenlet v. 0.4.15 grequests v. 0.3.0 grpcio v. 1.30.0 gseapy v. 0.9.13 h5py v. 2.10.0 hdmedians v. 0.13 HeapDict v. 1.0.0 html5lib v. 1.0.1 idna v. 2.8 imagesize v. 0.7.1 importlib-metadata v. 1.7.0 inotify v. 0.2.10 intervaltree v. 3.0.2 ipykernel v. 5.3.4 ipython v. 5.1.0 ipython-genutils v. 0.1.0 ipywidgets v. 7.5.1 itsdangerous v. 0.24 jdcal v. 1.2 jedi v. 0.9.0 Jinja2 v. 2.10.1 jmespath v. 0.9.4 joblib v. 0.16.0 jsonschema v. 2.5.1 jupyter v. 1.0.0 jupyter-client v. 6.1.3 jupyter-console v. 5.2.0 jupyter-contrib-core v. 0.3.3 jupyter-contrib-nbextensions v. 0.5.1 jupyter-core v. 4.6.3 jupyter-highlight-selected-word v. 0.2.0 jupyter-latex-envs v. 1.4.6 jupyter-nbextensions-configurator v. 0.4.1 kazoo v. 2.2.1 Keras-Preprocessing v. 1.1.2 keyring v. 10.6.0 keyrings.alt v. 3 kiwisolver v. 1.0.1 kneed v. 0.0.5 lazy-object-proxy v. 1.2.1 legacy-api-wrap v. 1.2 leidenalg v. 0.8.1 llvmlite v. 0.34.0 locket v. 0.2.0 lockfile v. 0.12.2 louvain v. 0.7.0 lxml v. 4.3.3 magic v. git+git://github.com/dpeerlab/magic.git@6f45a857ed9f9031b8ce3fd1fafb5b52d552fa99 Markdown v. 3.2.2 MarkupSafe v. 1.1.1 matplotlib v. 3.3.1 mistune v. 0.8.1

mock v. 2.0.0 mpmath v. 0.19 msgpack-python v. 0.4.8 MulticoreTSNE v. file:///opt/Multicore-TSNE multipledispatch v. 0.4.8 multiprocessing-on-dill v. 3.5.0a4 natsort v. 5.0.2 nbconvert v. 5.6.1 nbformat v. 5.0.6 nbpresent v. 3.0.0 networkx v. 2.5 nltk v. 3.2.1 nose v. 1.3.7 nose2 v. 0.9.1 notebook v. 6.1.1 numba v. 0.51.0 numexpr v. 2.6.9 numpy v. 1.16.0 numpy-indexed v. 0.3.4 oauthlib v. 3.1.0 odo v. 0.5.0 openpyxl v. 2.3.2 opt-einsum v. 3.3.0 packaging v. 20.4 palantir v. 0.2.6 pandas v. 1.0.0 pandocfilters v. 1.4.2 paramiko v. 2.4.2 parso v. 0.7.1 partd v. 0.3.6 path.py v. 2.2.2 pathlib2 v. 2.1.0 patsy v. 0.5.1 pbr v. 5.1.3 pdfrw v. 0.4 pep8 v. 1.7.0 pexpect v. 4.7.0 phate v. 0.2 PhenoGraph v. 1.5.6 pickleshare v. 0.7.4 Pillow v. 7.2.0 pkginfo v. 1.3.2 plotly v. 1.13.0 ply v. 3.9 prettytable v. 0.7.2 prometheus-client v. 0.8.0 prompt-toolkit v. 1.0.18 protobuf v. 3.12.2 psutil v. 4.3.1 ptyprocess v. 0.6.0 py v. 1.4.31 py-pcha v. 0.1.3 pyasn1 v. 0.4.6 pyasn1-modules v. 0.2.8 pycosat v. 0.6.1 pycparser v. 2.19 pycrypto v. 2.6.1 pyface v. 6.0.0 pyflakes v. 1.3.0 Pygments v. 2.1.3 pygobject v. 3.26.1 pylint v. 1.5.4 PyNaCl v. 1.3.0 pyOpenSSL v. 19.0.0 pyparsing v. 2.4.0 pypathway v. 0.2.4.2 Pyphen v. 0.9.5 pyrsistent v. 0.16.0 pysam v. 0.16.0.1 pytest v. 2.9.2 python-apt v. 1.6.5+ubuntu0.3 python-daemon-3K v. 1.5.8 python-dateutil v. 2.8.0 python-debian v. 0.1.32 python-igraph v. 0.8.2 python-json-logger v. 0.1.5 python-magic v. 0.4.18 pytz v. 2019.1 pyxdg v. 0.25 PyYAML v. 5.3.1 pyzmq v. 19.0.2 QtAwesome v. 0.3.3 qtconsole v. 4.7.4 QtPy v. 1.1.2 redis v. 2.10.5 regex v. 2019.4.10 reportlab v. 3.3.0 requests v. 2.21.0 requests-cache v. 0.4.13 requests-oauthlib v. 1.3.0 rope-py3k v. 0.9.4.post1 rsa v. 3.4.2 ruamel.yaml v. 0.16.10 ruamel.yaml.clib v. 0.2.0 s3transfer v. 0.3.0 scalpl v. 0.4.0 scanpy v. 1.4.6 scikit-bio v. 0.5.6 scikit-image v. 0.12.3 scikit-learn v. 0.23.2 scipy v. 1.4.1 scrublet v. scutils v. 1.1.0 seaborn v. 0.10.1 SecretStorage v. 2.3.1 Send2Trash v. 1.5.0 seqc v. 0.2.5 setuptools-scm v. 4.1.2 simplegeneric v. 0.8.1 singledispatch v. 3.4.0.3 six v. 1.12.0 sklearn v. 0 snowballstemmer v. 1.2.1 sortedcontainers v. 2.1.0 soupsieve v. 1.9 Sphinx v. 1.4.6 spyder v. 3.0.0 SQLAlchemy v. 1.0.13 ssh-import-id v. 5.7 statsmodels v. 0.12.0rc0 suds-jurko v. 0.6 sympy v. 1 tables v. 3.5.1 tensorboard v. 2.2.2 tensorboard-plugin-wit v. 1.7.0 tensorflow v. 2.2.0 tensorflow-estimator v. 2.2.0 tensorflow-probability v. 0.10.1 termcolor v. 1.1.0 terminado v. 0.8.3 testfixtures v. 4.13.3 testpath v. 0.4.4 texttable v. 1.6.2 threadpoolctl v. 2.1.0 tinycss2 v. 1.0.2 tinydb v. 3.13.0 toolz v. 0.8.0 tornado v. 5.1.1 tqdm v. 4.46.1 traitlets v. 4.3.3 traits v. 4.6.0 traitsui v. 5.1.0 tzlocal v. 2.1 umap-learn v. 0.4.6 urllib3 v. 1.24.1 uvloop v. 0.14.0 wcwidth v. 0.1.7 WeasyPrint v. 0.42.2 webencodings v. 0.5.1 Werkzeug v. 0.11.15 widgetsnbextension v. 3.5.1 wikipedia v. 1.4.0 wishbone v. 3.1.3 wrapt v. 1.11.1 xlrd v. 1.0.0 XlsxWriter v. 0.9.3 xlwt v. 1.1.2 xmltodict v. 0.12.0 zipp v. 3.1.0 zope.event v. 4.4 zope.interface v. 5.1.0

For manuscripts utilizing custom algorithms or software that are central to the research but not yet described in published literature, software must be made available to editors/reviewers. We strongly encourage code deposition in a community repository (e.g. GitHub). See the Nature Research guidelines for submitting code & software for further information.

## Data

Policy information about availability of data

All manuscripts must include a data availability statement. This statement should provide the following information, where applicable:

- Accession codes, unique identifiers, or web links for publicly available datasets
- A list of figures that have associated raw data
- A description of any restrictions on data availability

Data availability: The single cell RNA sequencing data generated in this study have been deposited in the NCBI's Gene Expression Omnibus database under accession code GSE160883 [https://www.ncbi.nlm.nih.gov/geo/query/acc.cgi?acc=GSE160883]. The processed single cell data and example notebooks are available at https://github.com/LaughneyLab/uveal-melanoma. The RNA and CUT&RUN sequencing data generated in this study have been deposited in the NCBI's Gene Expression Omnibus database under accession code GSE181600 [ [https://www.ncbi.nlm.nih.gov/geo/query/acc.cgi?acc=GSE181600]. The experimental data generated in this study (in figures 4b, 5c,d,f, 6a,b,d, 8a,b, and supplementary figures 6b, 8b,d,g-i, 9c-f, 10c, 11a and 11b) are provided in the Source Data file. Source data is provided with this paper. The following databases/datasets have been used in this study, KEGG database: https://www.genome.jp/kegg/; The validation single cell data cohort from Durante et. al 24 was accessed from NCBI's Gene Expression Omnibus database under accession code GSE139829 [https://www.ncbi.nlm.nih.gov/geo/query/acc.cgi?acc=GSE139829]. A ranked list of DEG and complete GSEA results per tumor archetype and cells analyzed in this manuscript are provided in Supplementary Data Files 2, 4-6. A custom GSEA annotation file, assembled to query cell types and pathways related to UM, PRC1/2 transcriptional signature and aneuploidy, as well as hallmark genesets is provided in Supplementary Data File 3.

# Field-specific reporting

Please select the one below that is the best fit for your research. If you are not sure, read the appropriate sections before making your selection.

☒ Life sciences ☐ Behavioural & social sciences ☐ Ecological, evolutionary & environmental sciences

For a reference copy of the document with all sections, see nature.com/documents/nr-reporting-summary-flat.pdf

# Life sciences study design

All studies must disclose on these points even when the disclosure is negative.

| | |
|---|---|
| Sample size | No sample size calculation was performed. Six enucleation specimens were obtained with at least one sample from each prognostic class based on bulk assessment from Castle Biosciences (GEP1 (n=2) and GEP2 (n=4)). For all experimental conditions, a minimum of biological or technical triplicates were used. |
| Data exclusions | No Data were excluded from the analysis |
| Replication | Experimental and biological replicates were done for all experiments as indicated in the figure legend. |
| Randomization | Not applicable to this study as there was no interventions |
| Blinding | Investigators were blinded to all experimental conditions including migration assays, growth assays, counting micronuclei, measuring nuclear size or counting patterns of chromosome missegregation. |

# Reporting for specific materials, systems and methods

We require information from authors about some types of materials, experimental systems and methods used in many studies. Here, indicate whether each material, system or method listed is relevant to your study. If you are not sure if a list item applies to your research, read the appropriate section before selecting a response.

## Materials & experimental systems

| n/a | Involved in the study |
| --- | --- |
| ☐ | ☒ Antibodies |
| ☐ | ☒ Eukaryotic cell lines |
| ☒ | ☐ Palaeontology |
| ☒ | ☐ Animals and other organisms |
| ☐ | ☒ Human research participants |
| ☒ | ☐ Clinical data |

## Methods

| n/a | Involved in the study |
| --- | --- |
| ☒ | ☐ ChIP-seq |
| ☒ | ☐ Flow cytometry |
| ☒ | ☐ MRI-based neuroimaging |

## Antibodies

Antibodies used

For immunofluorescence: Primary antibodies: centromere (Antibodies Incorporated, 15-234-0001), cGAS (Millipore, HPA031700) or Ubiquityl-H2AK119 (Cell Signaling, 8240) diluted in TBS-BSA 1:1600.

Western blots: BAP1 (Santa Cruz Biotechnology, sc-28383), 1:100; H2AK119Ub (Cell Signaling, 8240), 1:2000; RING1/RING1A (Cell Signaling, 2820), 1:250; RNF2/RING1B (Cell Signaling, 5694), STING (Cell Signaling, 13647), 1:1000; 1:250; Actin (Cell Signaling, 4970), 1:2000.

Corresponding HRP-conjugated secondary antibodies (mouse, Cell Signaling, 7076 and rabbit, Cell Signaling, 7074).

CUT&RUN: H2AK119Ub (Cell Signaling, 8240), 1:100; RING1 (Cell Signaling, 13069), 1:100; RNF2 (Cell Signaling, 5694), 1:100; BMI1 (Cell Signaling, 5856), 1:100; H3K27me3 (Thermo Fisher, MA5-11198), 1:100; IgG (Thermo Fisher, MA5-11198), 1:100.

Validation

Per manufacturers and cited papers. All antibodies in this study are validated by the manufacturer to be reactive to human proteins.

H3K27me3 (Thermo Fisher, MA5-11198). This antibody has been extensively validated for specificity using SNAP-ChIP™ spike-in and peptide array. This Antibody was verified by Peptide array to ensure that the antibody binds to the antigen stated.

H2AK119Ub (Cell Signaling, 8240) - This antibody has been validated using SimpleChIP® Enzymatic Chromatin IP Kits. (total 163 citations)

RING1/RING1A (Cell Signaling, 2820) - 10 citations

RNF2/RING1B (Cell Signaling, 5694) - 48 citations - This antibody has been validated using SimpleChIP® Enzymatic Chromatin IP Kits.

Actin (Cell Signaling, 4970) - 2918 citations

BMI (Cell Signaling, 5856) - This antibody has been validated using SimpleChIP® Enzymatic Chromatin IP Kits. 26 citations

cGAS (Millipore, HPA031700)

STING (Cell Signaling, 13647) 157 citations - The antibody has been carefully validated in-house (Cell Signaling) and is working well for Western blot, Immunoprecipitation, and IHC using their recommended protocols.

## Eukaryotic cell lines

Policy information about cell lines

Cell line source(s)

HEK293T and established human UM cell lines MP41, MP38, MP46 and Mel285 were purchased from the American Type Culture Collection (ATCC), and 92.1 and Mel202 from the European Collection of Cell Cultures (ECACC).

Authentication

The cell lines were authenticated by ATCC and ECACC. BAP1 expression was confirmed by the investigators in all cell lines. No additional authentication was performed.

Mycoplasma contamination

Mycoplasma testing was performed by ATCC and ECACC. All cell lines were tested every 3 months and shown to be negative for mycoplasma.

Commonly misidentified lines
(See ICLAC register)

None

## Human research participants

Policy information about studies involving human research participants

| | |
|---|---|
| Population characteristics | Tumors were obtained from enucleation specimens from six participants. The demographics are shown in Extended Data Table 1. |
| Recruitment | Patients with uveal melanoma undergoing enucleation were recruited to the study. Patients with small tumors often elect to undergo local radiotherapy. Hence, there is a selection bias towards larger tumors. Since the majority were from larger tumors there is an increased proportion of GEP2 cells. |
| Ethics oversight | All protocols adhered to the tenets of the Declaration of Helsinki and were conducted in accordance with the regulations of the Health Insurance Portability and Accountability Act. Internal Review Board (IRB) approval was obtained from Memorial Sloan Kettering Cancer Center, NY (IRB protocol #17-206). |

Note that full information on the approval of the study protocol must also be provided in the manuscript.
